# Supplementary material for: New Cytotoxic Cytochalasans from a Plant-Associated Fungus Chaetomium globosum kz-19
Source: Mar Drugs. 2021 Jul 31;19(8):438. doi: 10.3390/md19080438 (PMC8398356; doi:10.3390/md19080438)

Supporting Information

New Cytotoxic Cytochalasans from A Plant-Associated Fungus *Chaetomium globosum* kz-19

Tantan Li[1,2], Yun Wang[1], Li Li[4], Mengyue Tang[1,3], Qinghong Meng[1,2], Cun Zhang[1], Erbing Hua[2,*], Yuehu Pei[3,*], Yi Sun[1,*]

[1] Institute of Chinese Materia Medica, China Academy of Chinese Medical Sciences, Beijing 100700, PR China; tantanna0309@163.com (T.L.); uwang@cimm.ac.cn (Y.W.); qinghongmeng0813@hotmail.com (Q.M.); czhang@icmm.ac.cn (C.Z.)

[2] College of Biotechnology, Tianjin University of Science & Technology, Tianjin 300457, PR China;

[3] School of Pharmacy, Harbin Medical University, Harbin 150081, PR China;

[4] Institute of Materia Medica, Chinese Academy of Medical Sciences & Peking Union Medical College, Beijing 100050, PR China; annaleelin@imm.ac.cn (L.L.)

* Correspondence: peiyueh@vip.163.com (Y. P.); huarb@ tust.edu.cn (E.H.); ysun@icmm.ac.cn (Y.S.); Tel.: +86-10-6403-2656 (Y.S.)

Contents	Page
Figure S1: ^{1}H NMR (600 MHz, DMSO-*D6*) spectrum of **1**	3
Figure S2: ^{13}C NMR (150 MHz, DMSO-*D6*) spectrum of **1**	3
Figure S3: HSQC spectrum of **1**	4
Figure S4: HMBC spectrum of **1**	4
Figure S5: ^{1}H-^{1}H COSY spectrum of **1**	5
Figure S6: TOCSY spectrum of **1**	5
Figure S7: NOESY spectrum of **1**	6
Figure S8: HRESI-MS spectrum of **1**	6
Figure S9: ECD and UV spectrum of **1**	6
Figure S10: ^{1}H NMR (600 MHz, DMSO-*D6*) spectrum of **2**	7
Figure S11: ^{13}C NMR (150 MHz, DMSO-*D6*) spectrum of **2**	7
Figure S12: HSQC spectrum of **2**	8
Figure S13: HMBC spectrum of **2**	8
Figure S14: ^{1}H-^{1}H COSY spectrum of **2**	9
Figure S15: TOCSY spectrum of **2**	9
Figure S16: NOESY spectrum of **2**	10
Figure S17: HRESI-MS spectrum of **2**	10
Figure S18: ECD and UV spectrum of **2**	10-11
Figure S19: ^{1}H NMR (600 MHz, DMSO-*D6*) spectrum of **2c**	12
Figure S20: ^{1}H-^{1}H COSY spectrum of **2c**	12
Figure S21: ^{1}H NMR (600 MHz, DMSO-*D6*) spectrum of **2d**	13
Figure S22: ^{1}H-^{1}H COSY spectrum of **2d**	13
Figure S23: ^{1}H NMR (600 MHz, DMSO-*D6*) spectrum of **3**	14
Figure S24: ^{13}C NMR (150 MHz, DMSO-*D6*) spectrum of **3**	14
Figure S25: HSQC spectrum of **3**	15
Figure S26: HMBC spectrum of **3**	15
Figure S27: ^{1}H-^{1}H COSY spectrum of **3**	16
Figure S28: ROESY spectrum of **3**	16
Figure S29: HRESI-MS spectrum of **3**	17
Figure S30: ECD and UV spectrum of **3**	17
Figure S31: ^{1}H NMR (600 MHz, DMSO-*D6*) spectrum of **4**	18
Figure S32: ^{13}C NMR (150 MHz, DMSO-*D6*) spectrum of **4**	18
Figure S33: HSQC spectrum of **4**	19
Figure S34: HMBC spectrum of **4**	19
Figure S35: ^{1}H-^{1}H COSY spectrum of **4**	20
Figure S36: TOCSY spectrum of **4**	20
Figure S37: NOESY spectrum of **4**	21
Figure S38: HRESI-MS spectrum of **4**	21
Figure S39: ECD and UV spectrum of **4**	22
Figure S40: ^{1}H NMR (600 MHz, DMSO-*D6*) spectrum of **5**	23
Figure S41: ^{13}C NMR (150 MHz, DMSO-*D6*) spectrum of **5**	22
Figure S42: ^{1}H NMR (600 MHz, DMSO-*D6*) spectrum of **6**	23
Figure S43: ^{13}C NMR (150 MHz, DMSO-*D6*) spectrum of **6**	23

Figure S44: ^{1}H NMR (600 MHz, DMSO-*D6*) spectrum of **7**	24
Figure S45: ^{13}C NMR (150 MHz, DMSO-*D6*) spectrum of **7**	24
Figure S46: ^{1}H NMR (600 MHz, DMSO-*D6*) spectrum of **8**	25
Figure S47: ^{13}C NMR (150 MHz, DMSO-*D6*) spectrum of **8**	25
Figure S48: ^{1}H NMR (600 MHz, DMSO-*D6*) spectrum of **9**	26
Figure S49: ^{13}C NMR (150 MHz, DMSO-*D6*) spectrum of **9**	26
Figure S50: ^{1}H NMR (600 MHz, DMSO-*D6*) spectrum of **10**	27
Figure S51: ^{13}C NMR (150 MHz, DMSO-*D6*) spectrum of **10**	27
Figure S52: ^{1}H NMR (600 MHz, DMSO-*D6*) spectrum of **11**	28
Figure S53: ^{13}C NMR (150 MHz, DMSO-*D6*) spectrum of **11**	28
Figure S54: ^{1}H NMR (600 MHz, DMSO-*D6*) spectrum of **12**	29
Figure S55: ^{13}C NMR (150 MHz, DMSO-*D6*) spectrum of **12**	29
Figure S56: ^{1}H NMR (600 MHz, DMSO-*D6*) spectrum of **13**	30
Figure S57: ^{13}C NMR (150 MHz, DMSO-*D6*) spectrum of **13**	30
Figure S58: ^{1}H NMR (600 MHz, DMSO-*D6*) spectrum of **14**	31
Figure S59: ^{13}C NMR (150 MHz, DMSO-*D6*) spectrum of **14**	31
Figure S60: ^{1}H NMR (600 MHz, DMSO-*D6*) spectrum of **15**	32
Figure S61: ^{13}C NMR (150 MHz, DMSO-*D6*) spectrum of **15**	32
Figure S62: ^{1}H NMR (600 MHz, DMSO-*D6*) spectrum of **16**	33
Figure S63: ^{13}C NMR (150 MHz, DMSO-*D6*) spectrum of **16**	33

Figure S1: ^{1}H NMR (600 MHz, DMSO-*D6*) spectrum of **1**

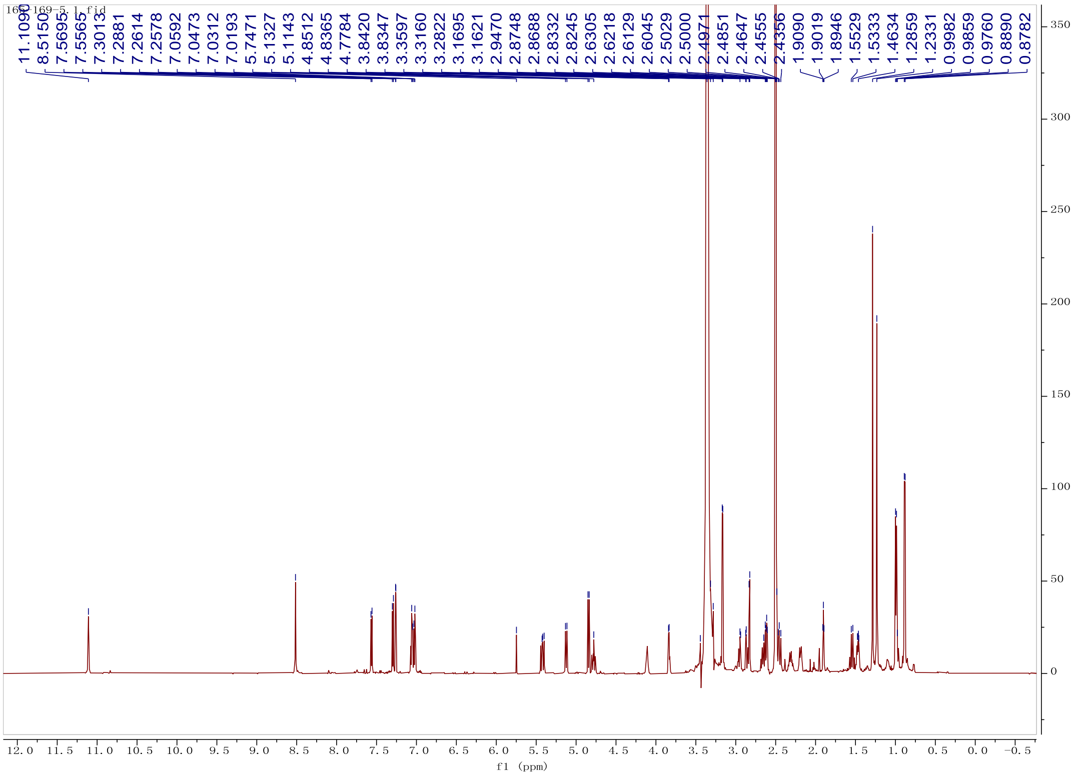

Figure S2: ^{13}C NMR (150 MHz, DMSO-*D6*) spectrum of **1**

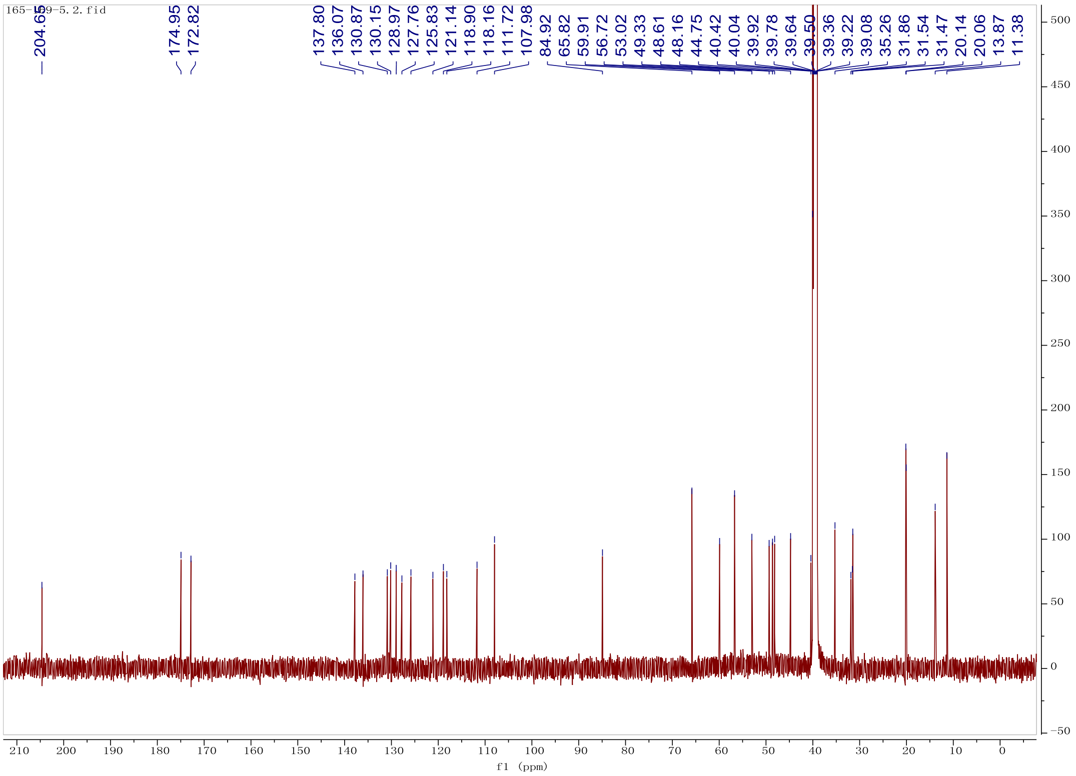

Figure S3: HSQC spectrum of **1**

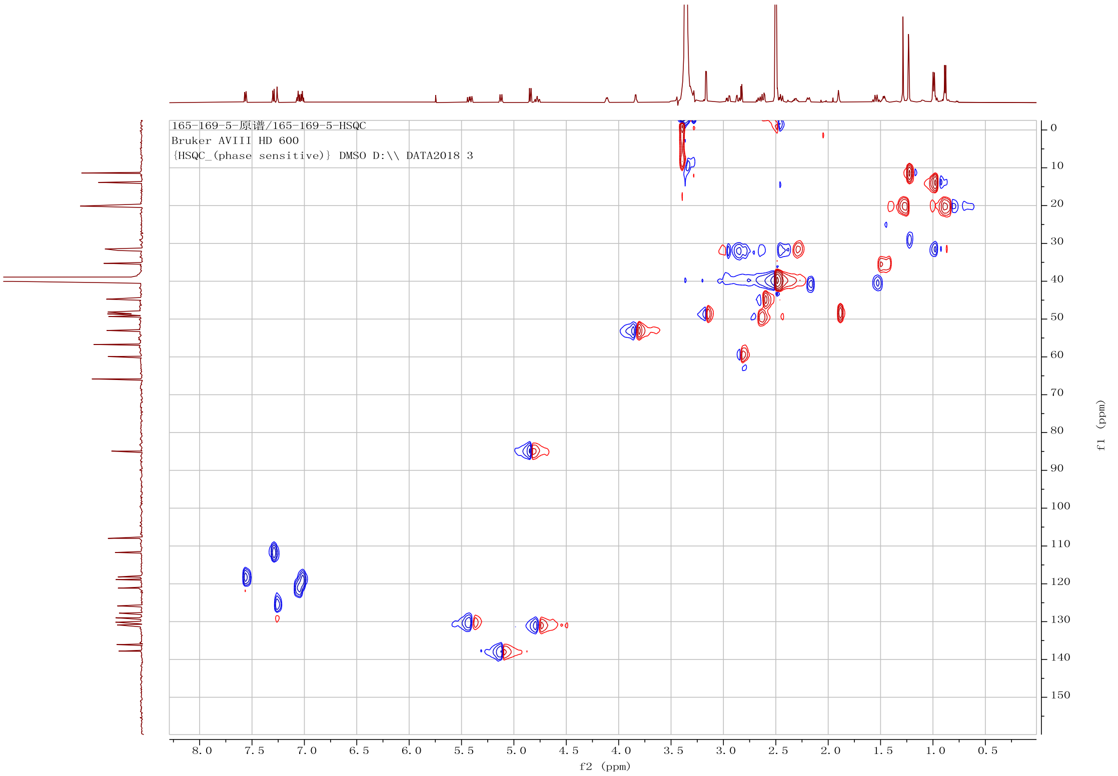

Figure S4: HMBC spectrum of **1**

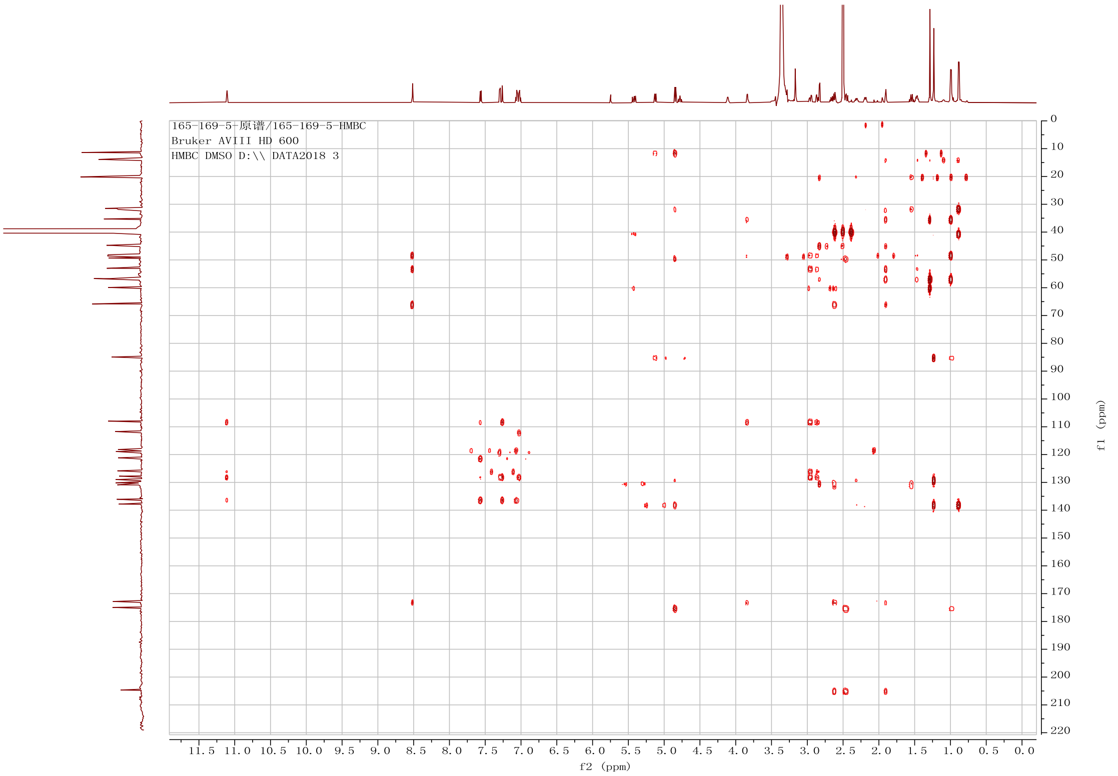

Figure S5: ^{1}H-^{1}H COSY spectrum of **1**

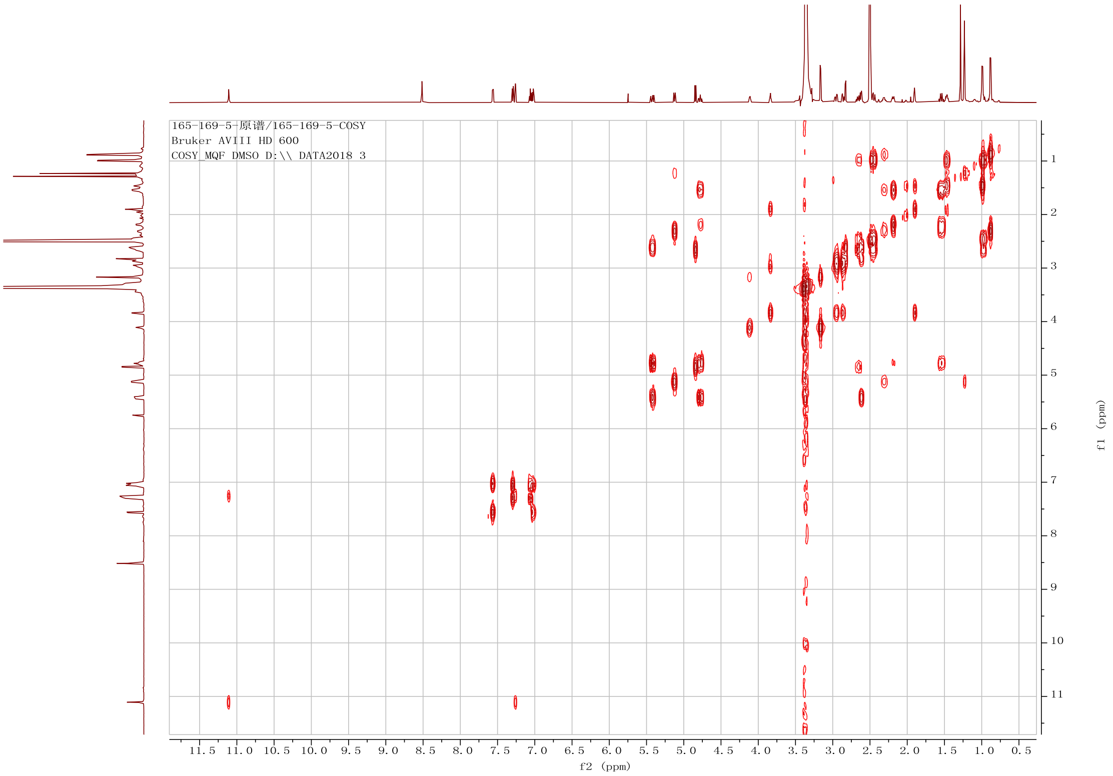

Figure S6: TOCSY spectrum of **1**

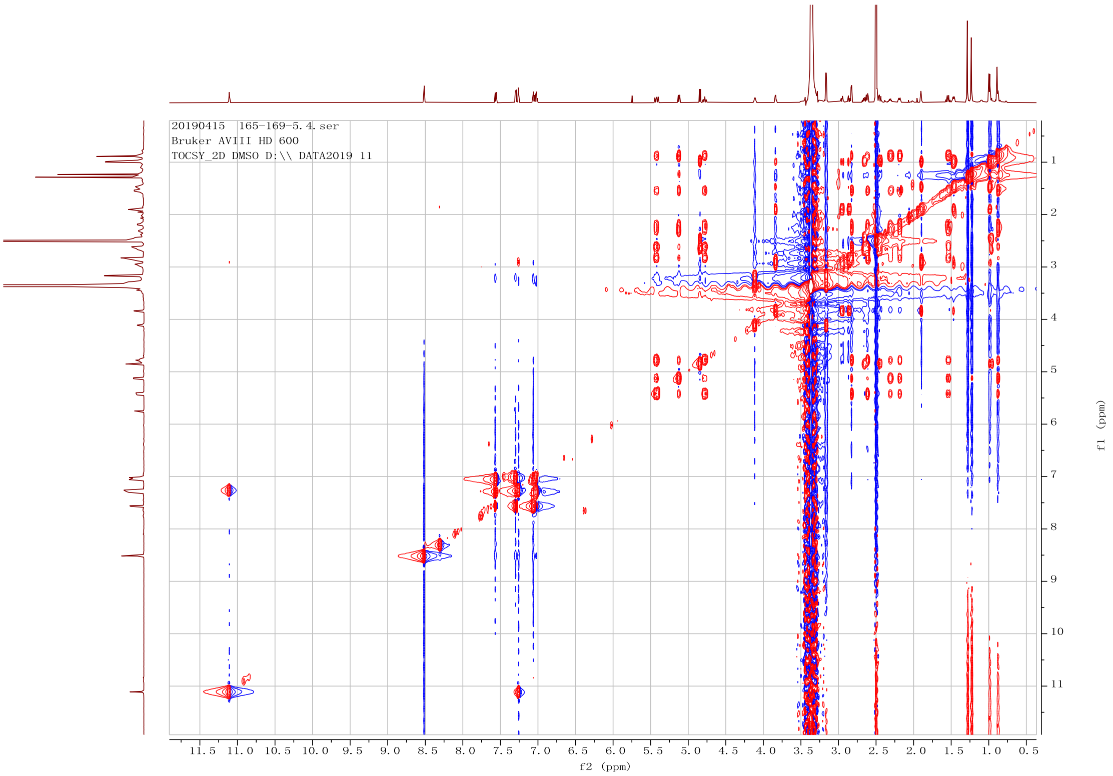

Figure S7: NOESY spectrum of **1**

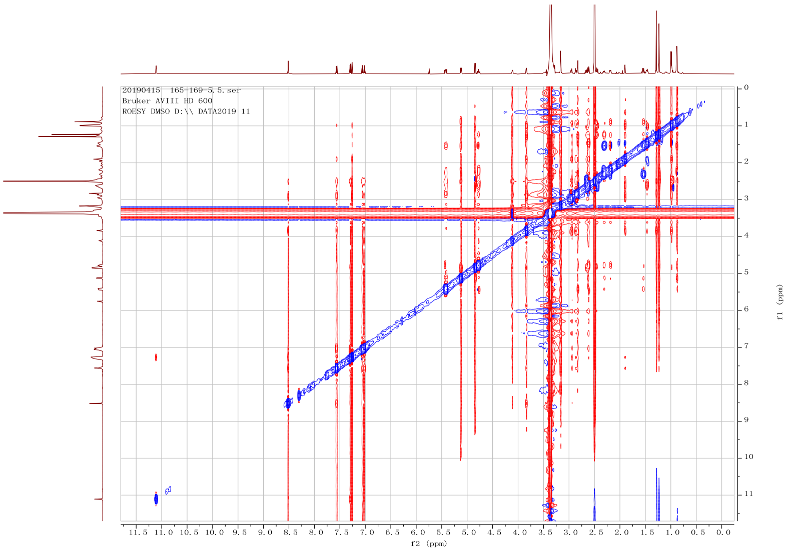

Figure S8: HR ESIMS spectrum of **1**

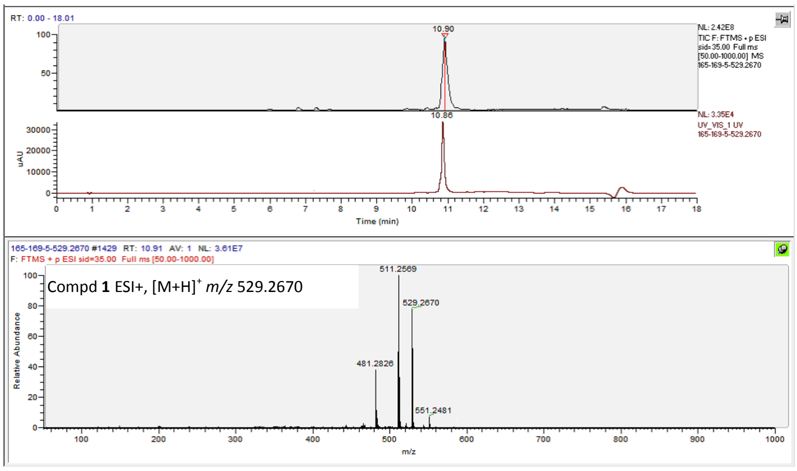

Figure S9: ECD and UV spectrum of **1**

TDDFT theory, ωB3LYP functional and 6-311G(d,p) level of theory, methanol as solvent for structural optimization, compound **1** have 9 conformations, of which 2 conformations have Boltzmann content >1%, listed in the table. The calculated result of **1** is consistent with the experimental result, and the absolute configuration of **1** is confirmed as shown in the figure below (σ=0.30eV).

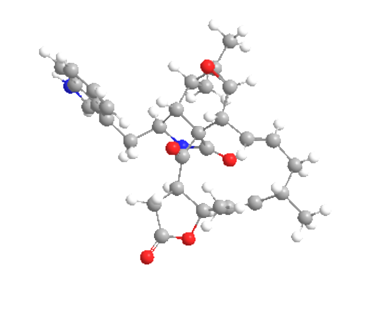	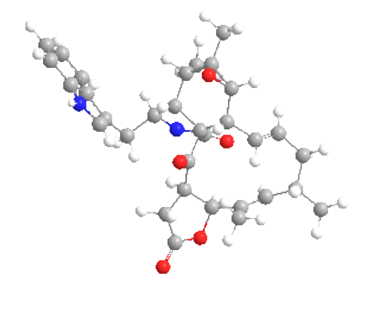
**1a** (92.55%)	**1b** (6.92%)

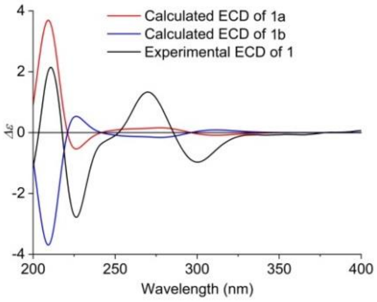

Figure S10: ^{1}H NMR (600 MHz, DMSO-*D6*) spectrum of **2**

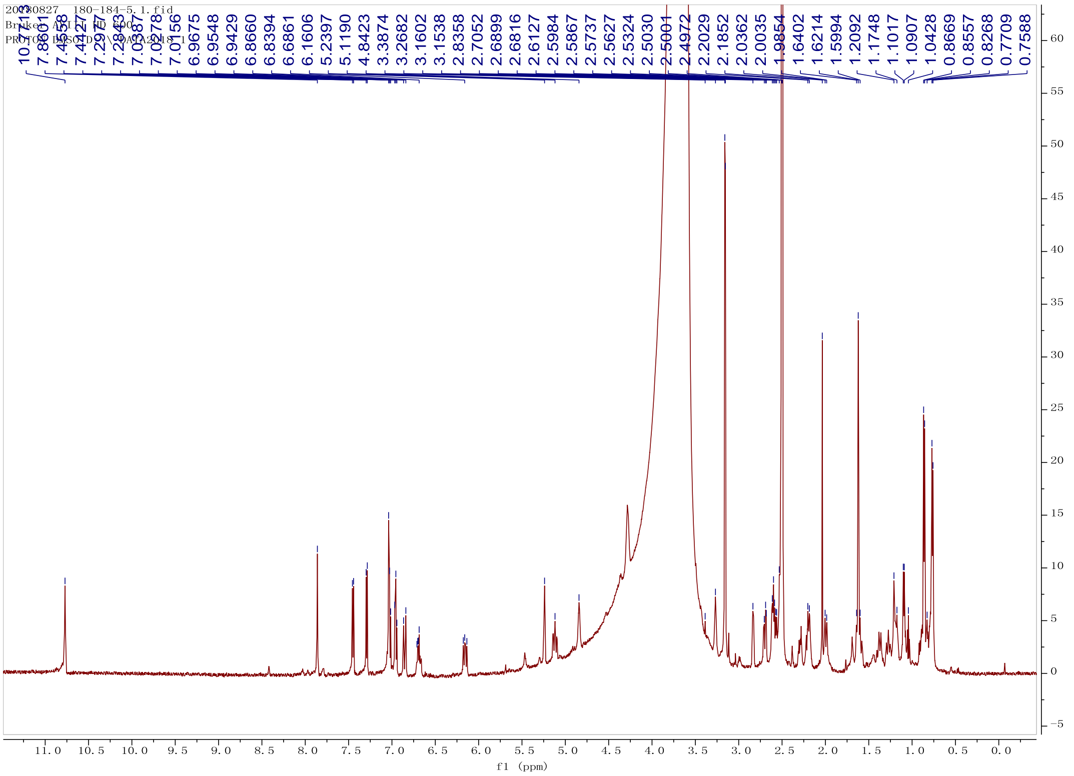

Figure S11: ^{13}C NMR (150 MHz, DMSO-*D6*) spectrum of **2**

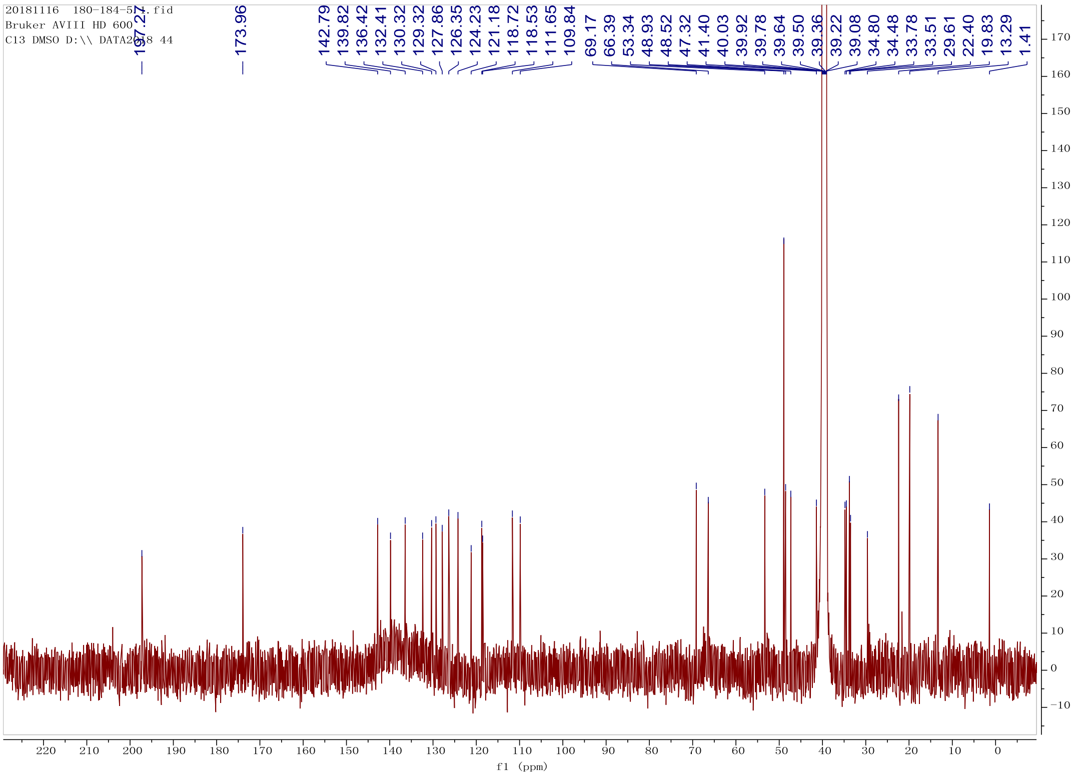

Figure S12: HSQC spectrum of **2**

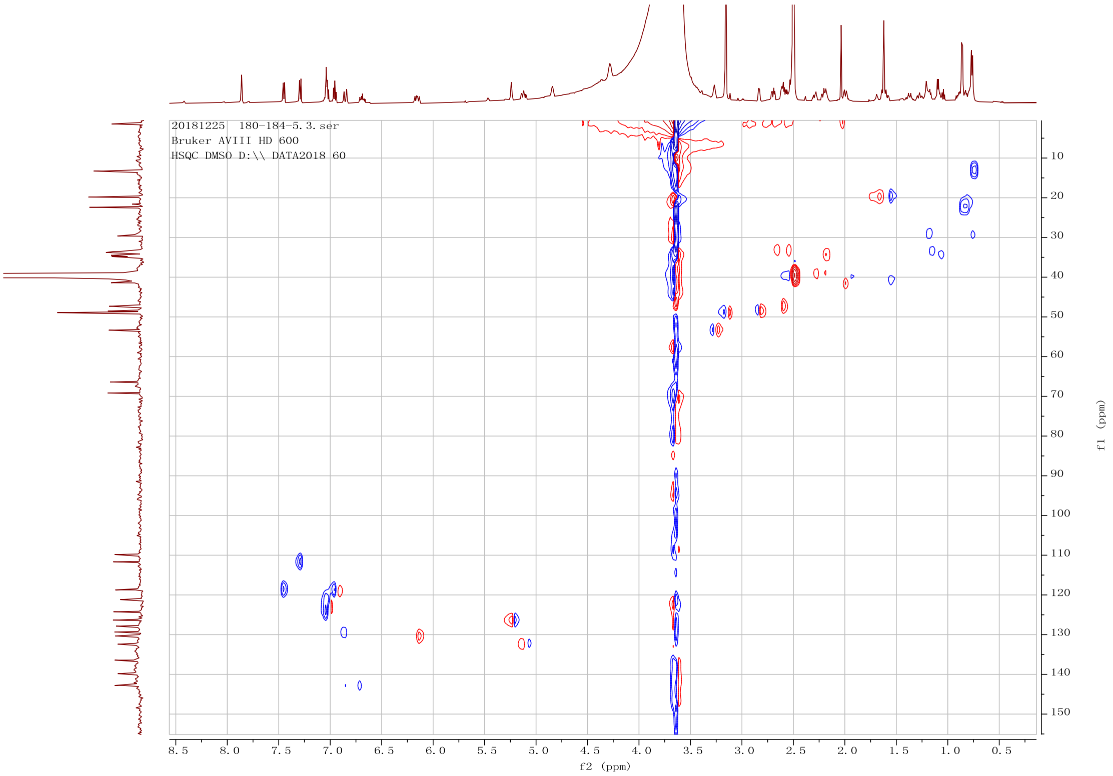

Figure S13: HMBC spectrum of **2**

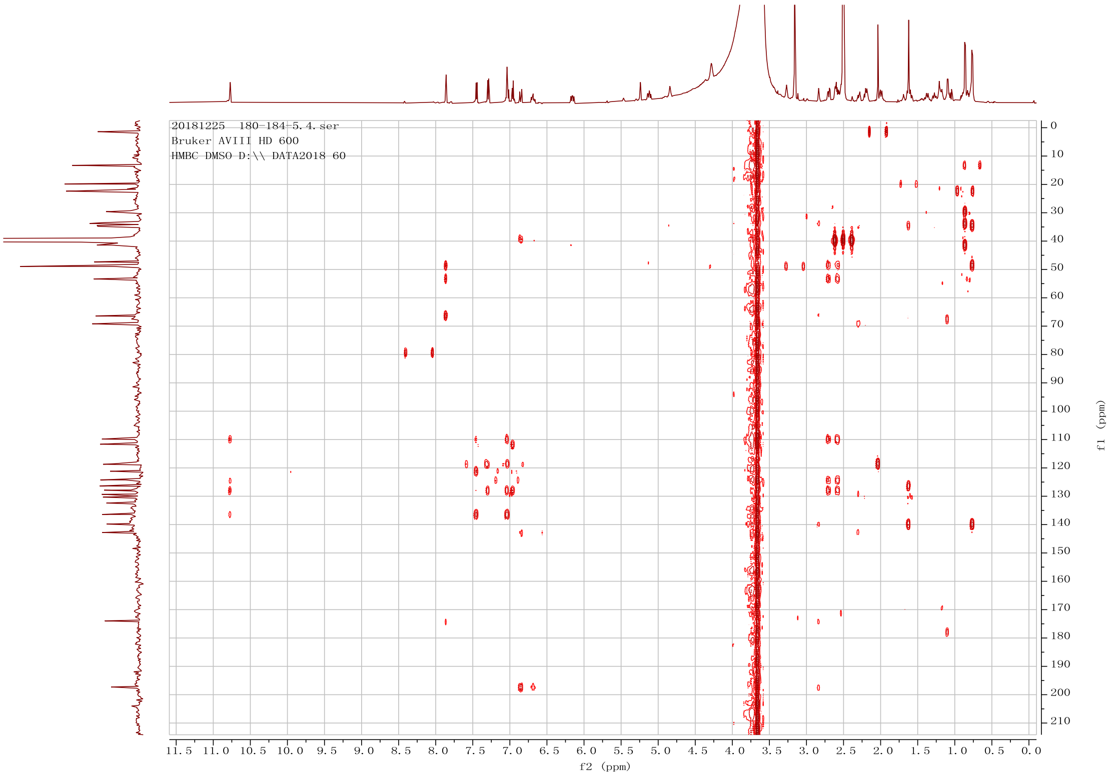

Figure S14: ^{1}H-^{1}H COSY spectrum of **2**

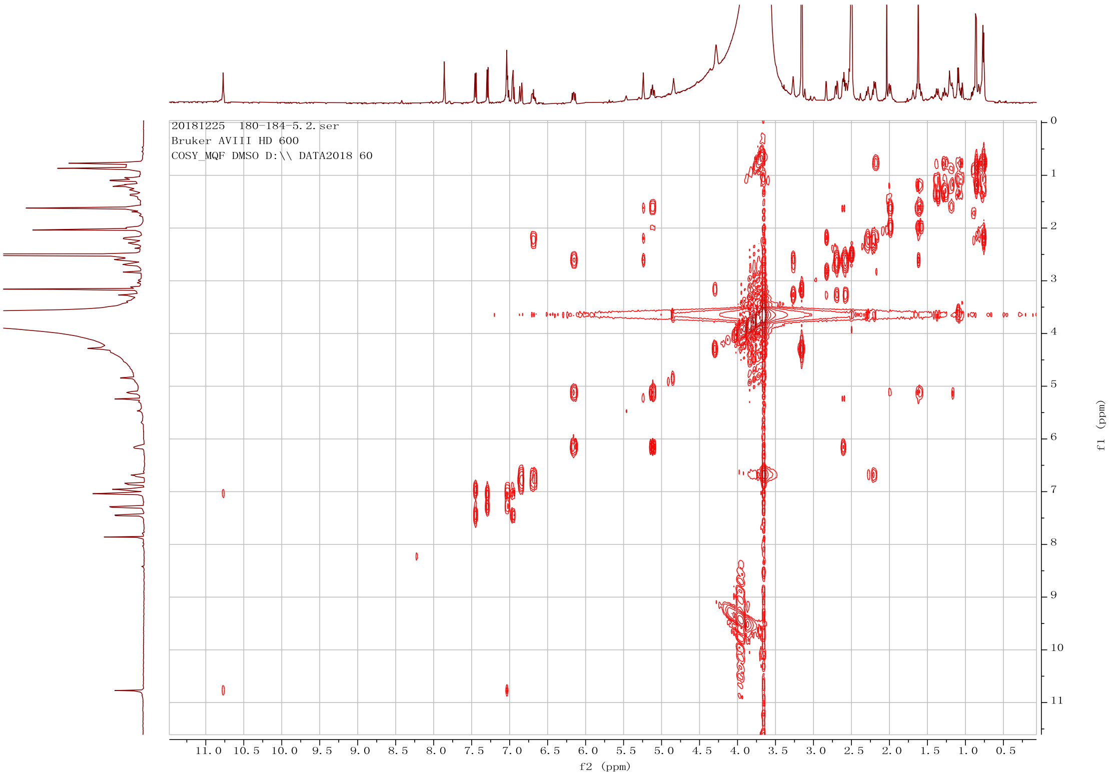

Figure S15: TOCSY spectrum of **2**

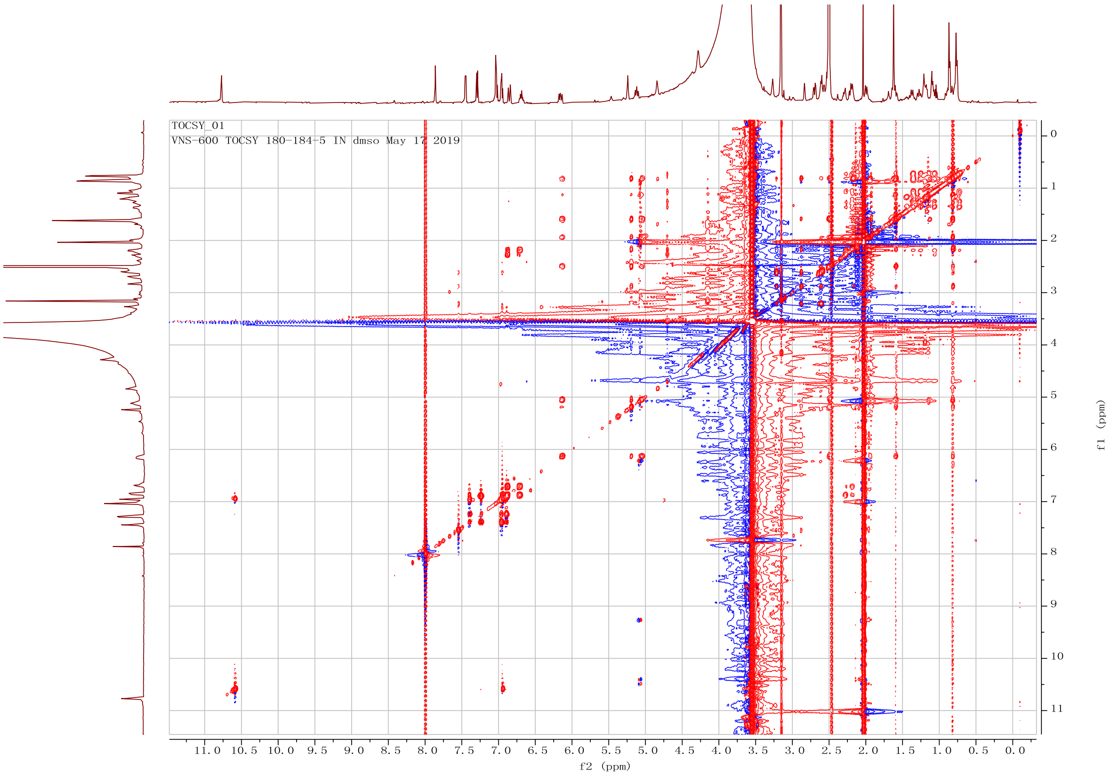

Figure S16: NOESY spectrum of **2**

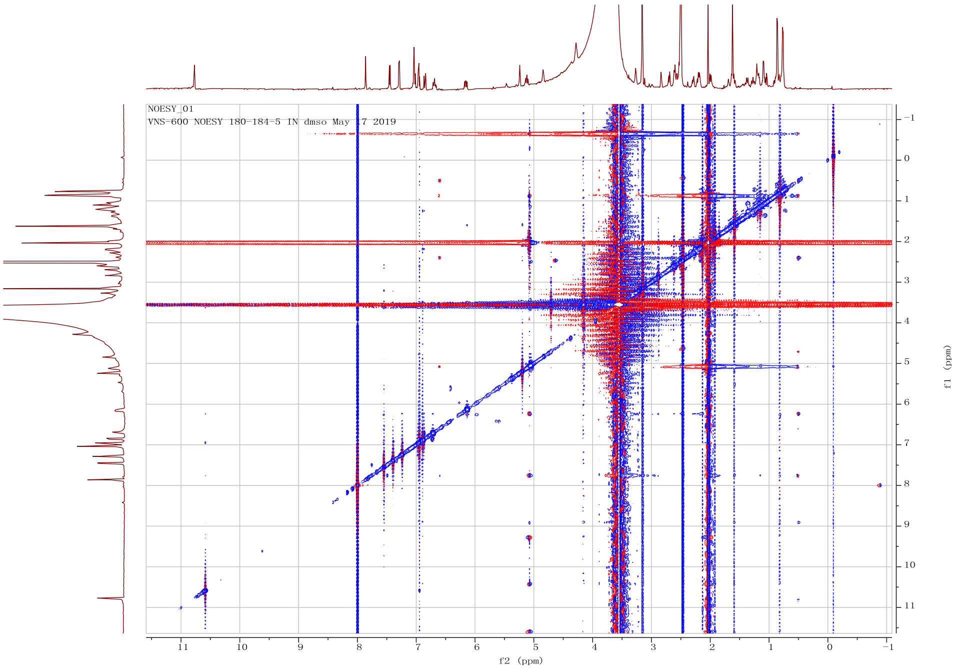

Figure S17: HR ESIMS spectrum of **2**

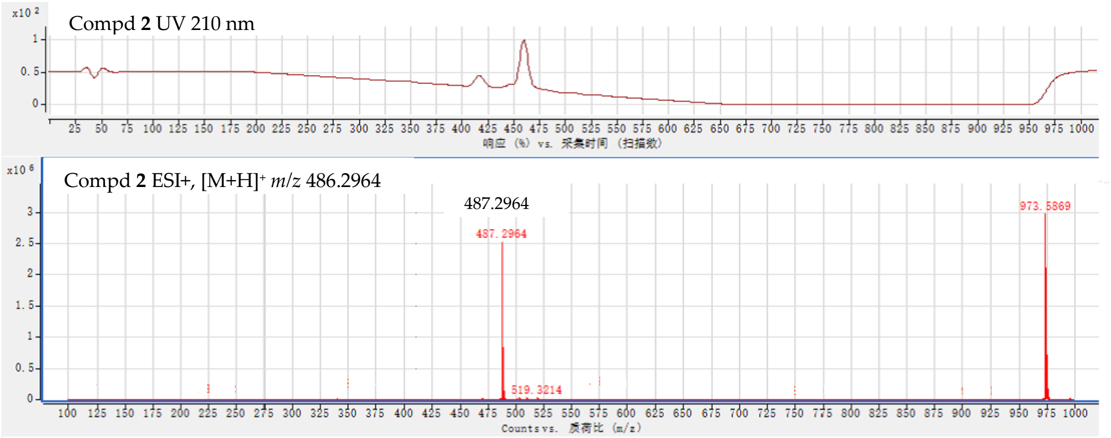

Figure S18: ECD and UV spectrum of **2**

TDDFT theory, ωB97XD functional and TZVP level of theory, **2** has 58 conformations, of which 14 conformations have Boltzmann content >1%, listed in the table below. Cam-B3LYP functional and TZVP level calculate 70 excited states, the solvent is methanol, σ=0.40eV, the result is shown in the figure.

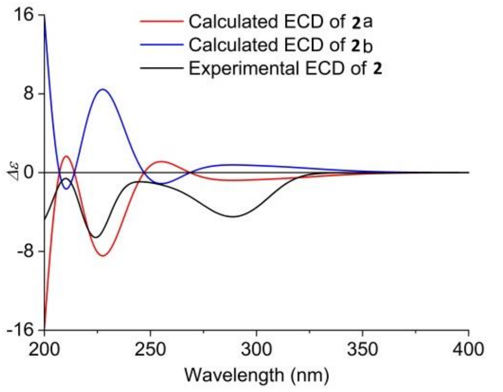

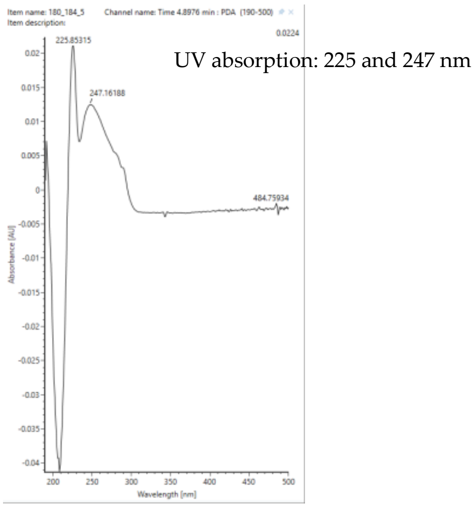

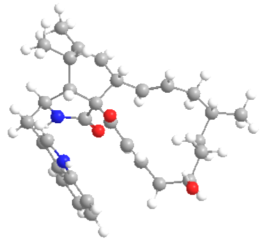	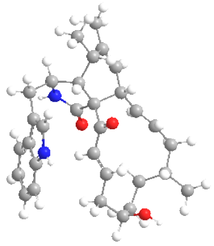	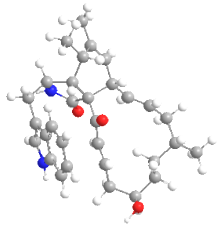
C1 (16.70%)	C2 (16.18%)	C3 (11.95%)
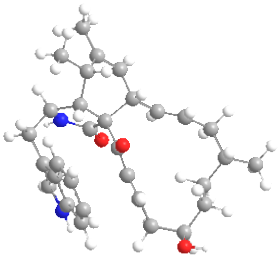	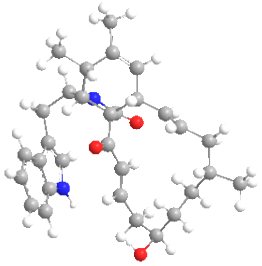	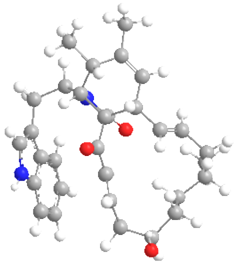
C4 (9.76%)	C5 (9.21%)	C6 (8.23%)
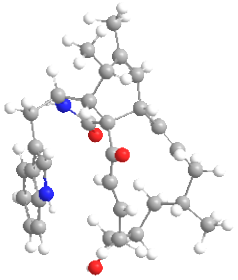	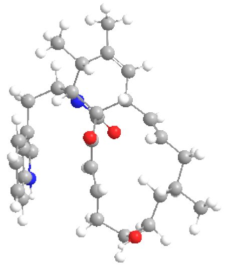	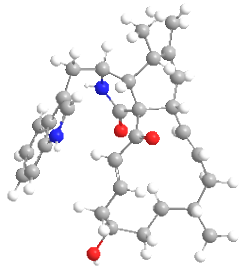
C7 (7.77%)	C8 (4.31%)	C9 (3.55%)
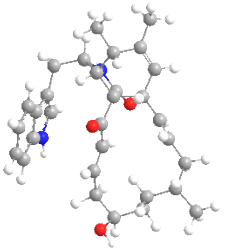	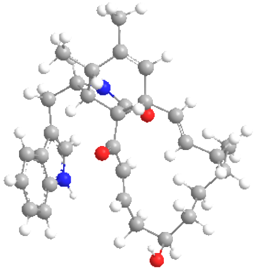	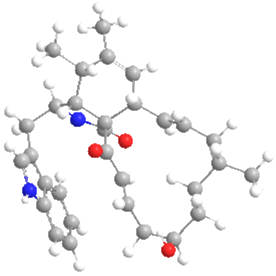
C10 (3.04%)	C11 (2.29%)	C12 (2.20%)
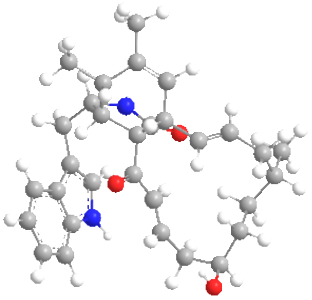	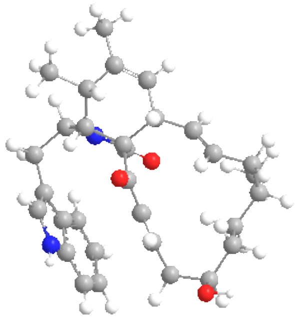	
C13 (2.01%)	C14 (1.20%)	

Figure S19: ^{1}H NMR spectrum of **2c**

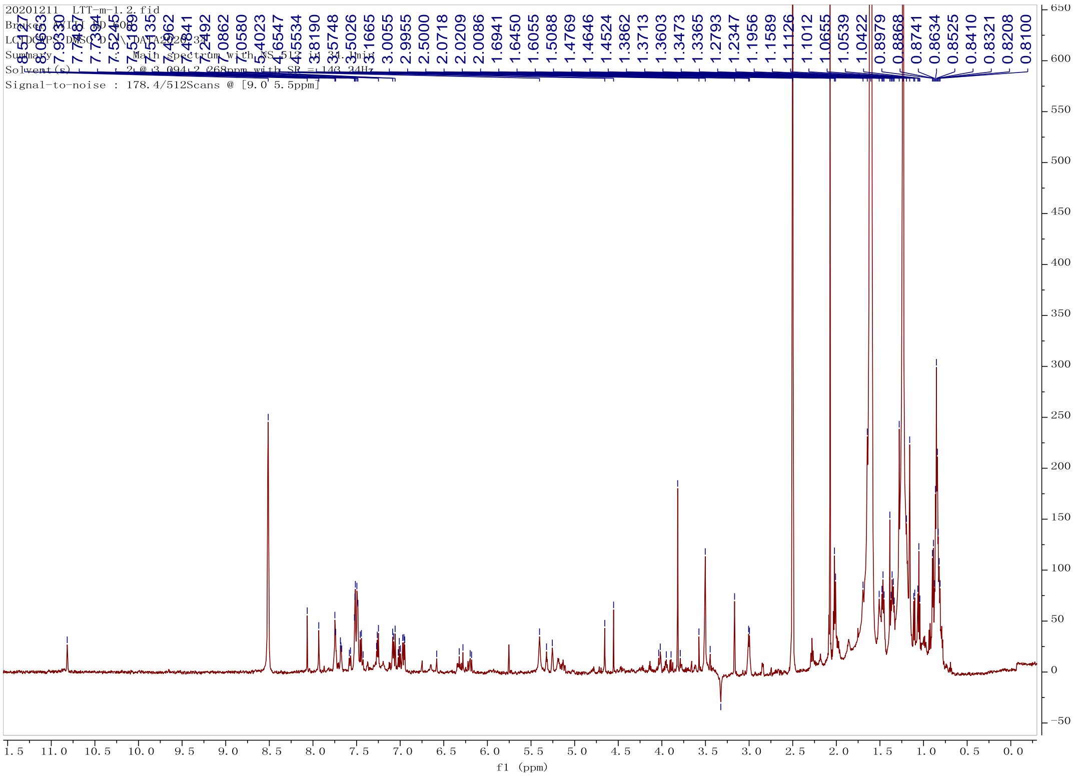

Figure S20: ^{1}H-^{1}H COSY spectrum of **2c**

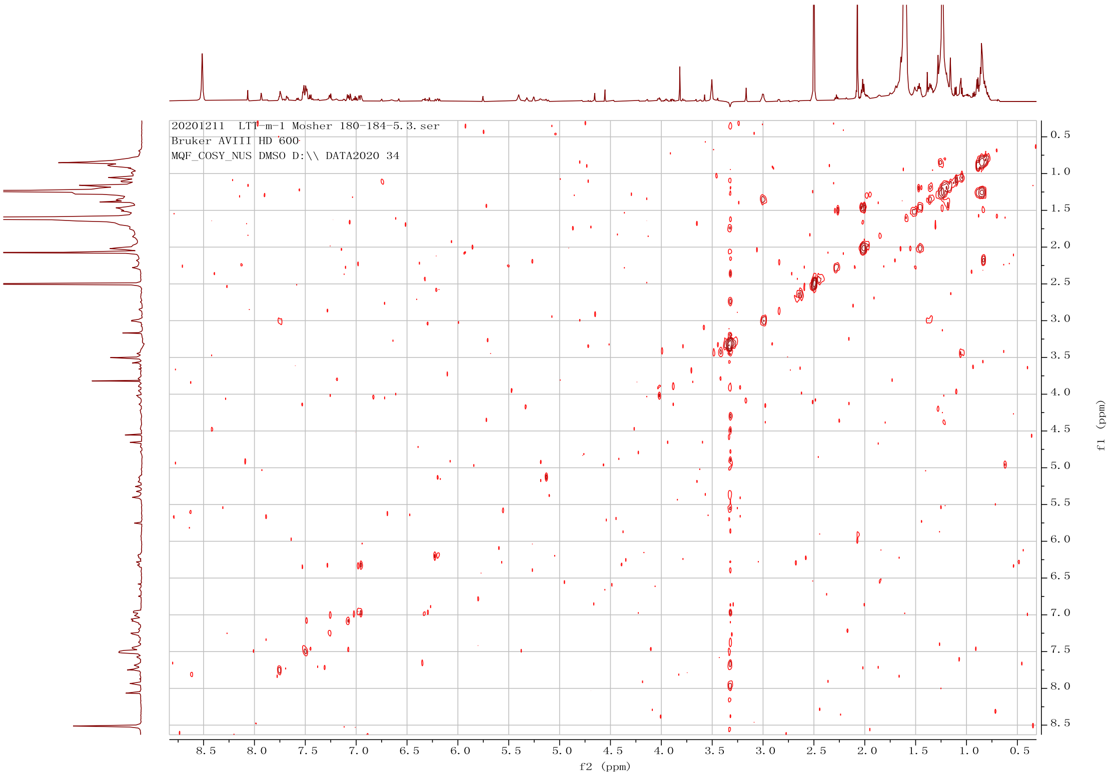

Figure S21: ^{1}H-^{1}H COSY spectrum of **2d**

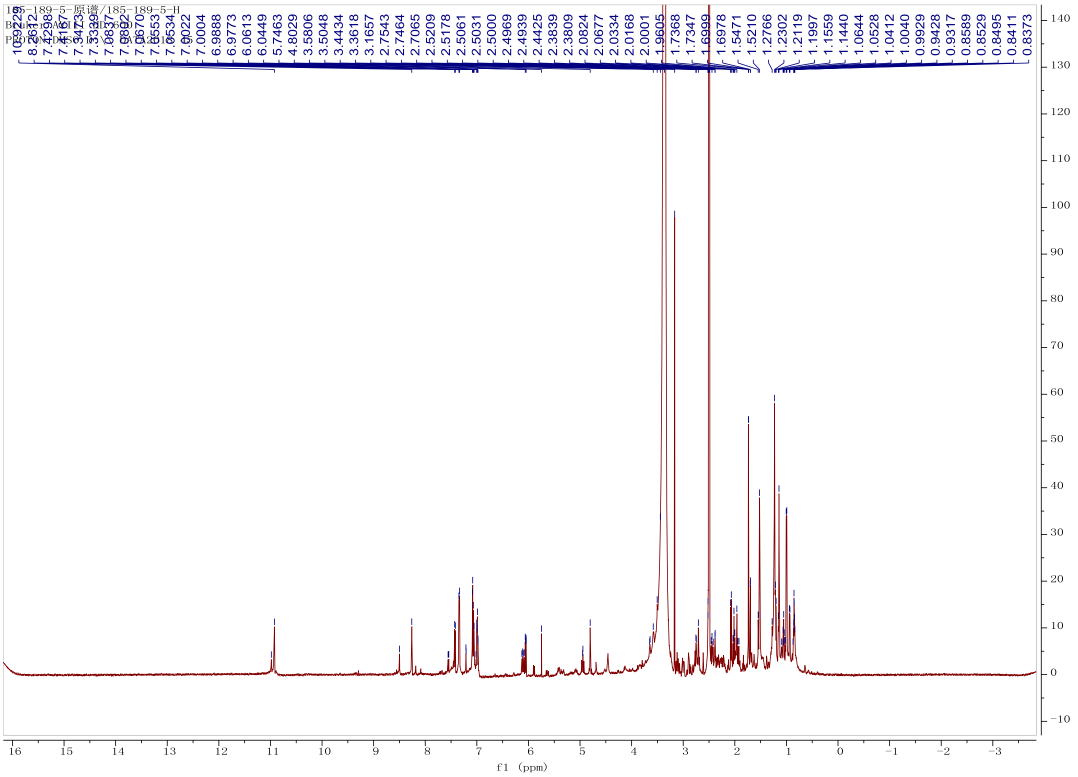

Figure S22: ^{1}H-^{1}H COSY spectrum of **2d**

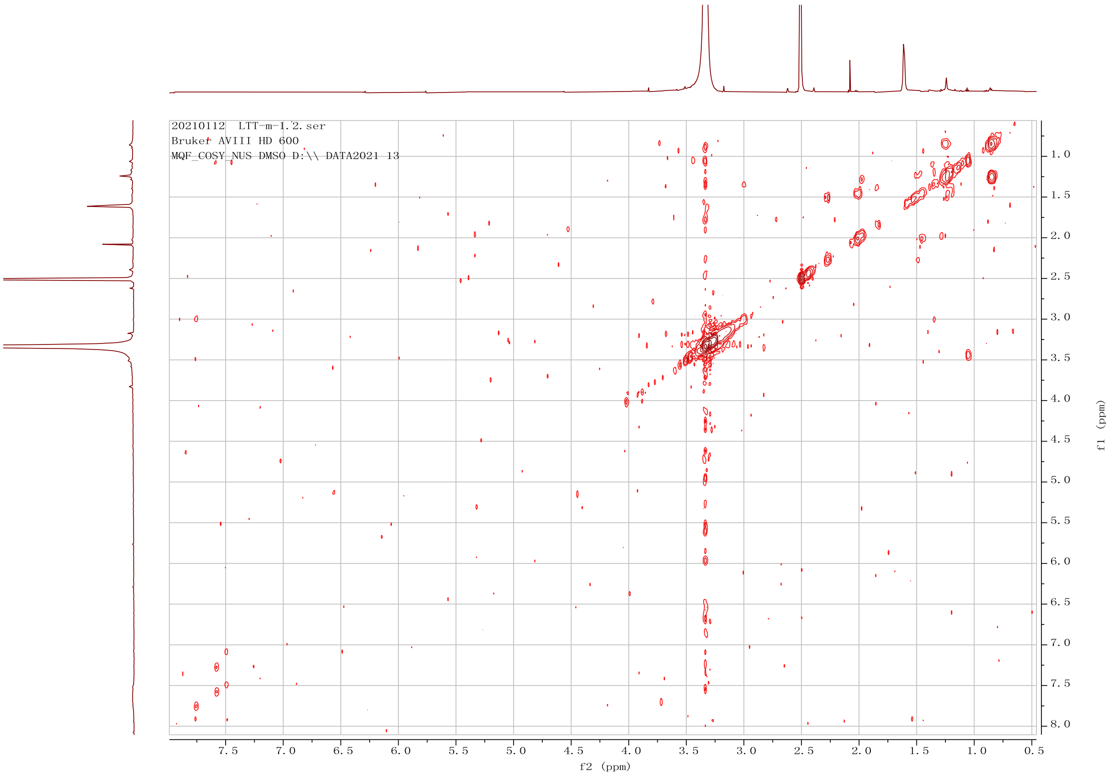

Figure S23: ^{1}H NMR (600 MHz, DMSO-*D6*) spectrum of **3**

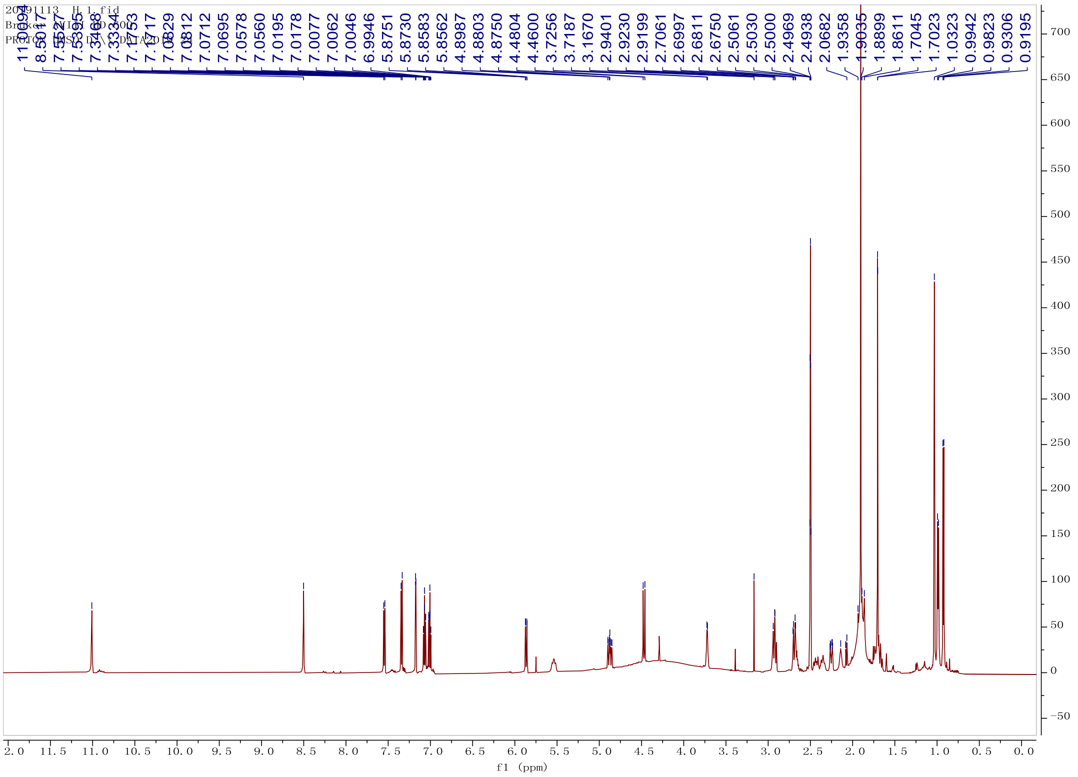

Figure S24: ^{13}C NMR (150 MHz, DMSO-*D6*) spectrum of **3**

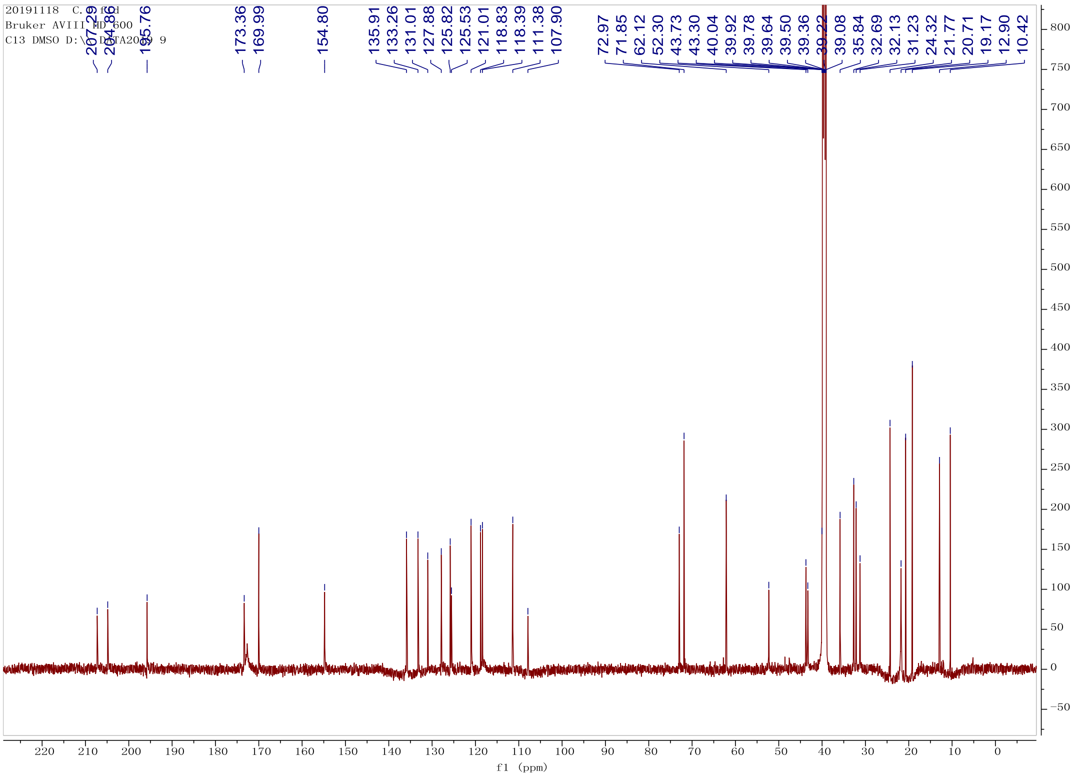

Figure S25: HSQC spectrum of **3**

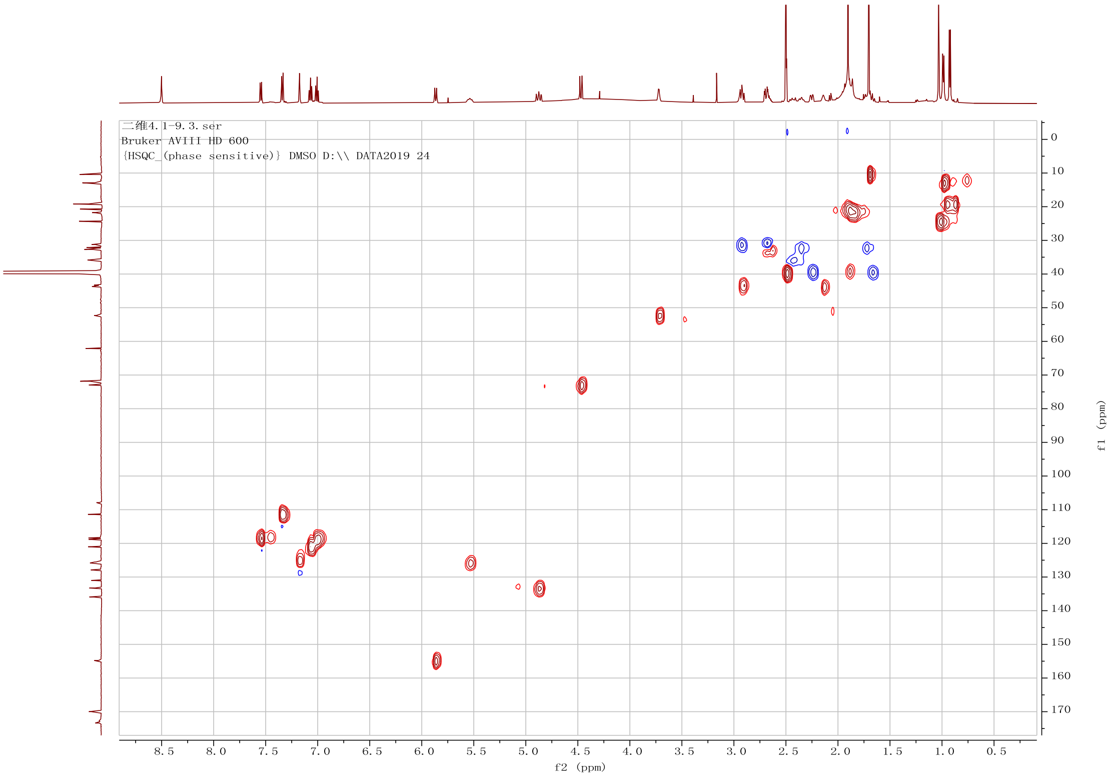

Figure S26: HMBC spectrum of **3**

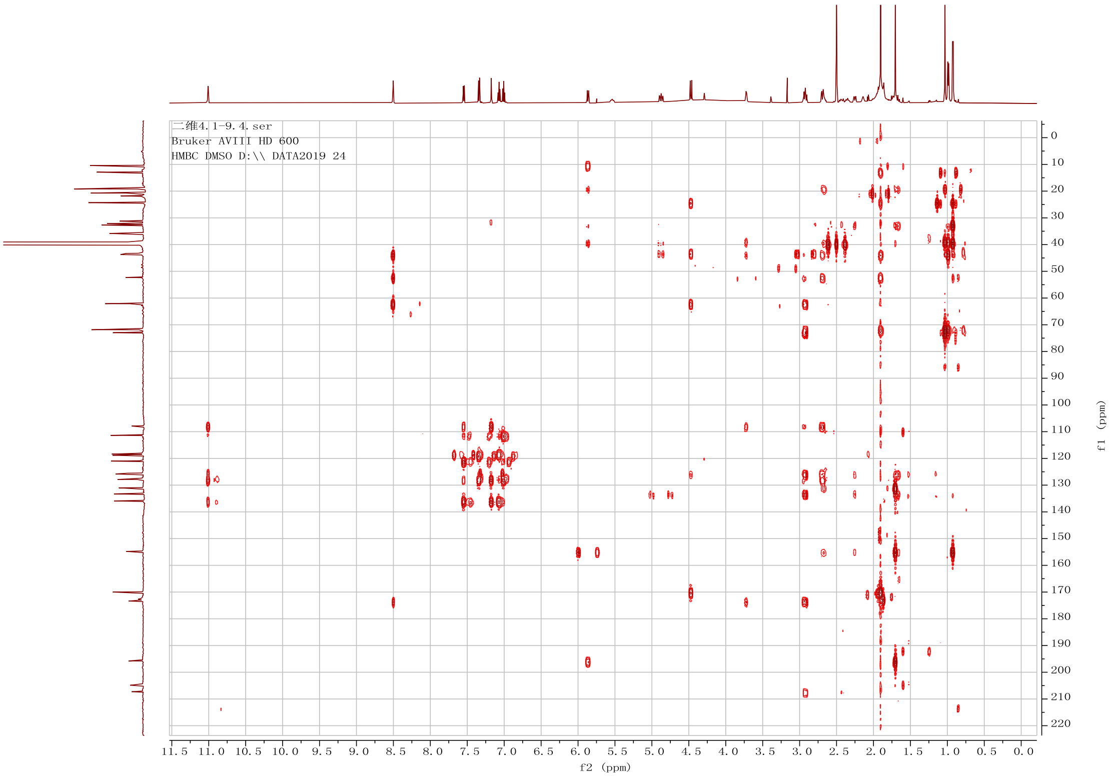

Figure S27: ^{1}H-^{1}H COSY spectrum of **3**

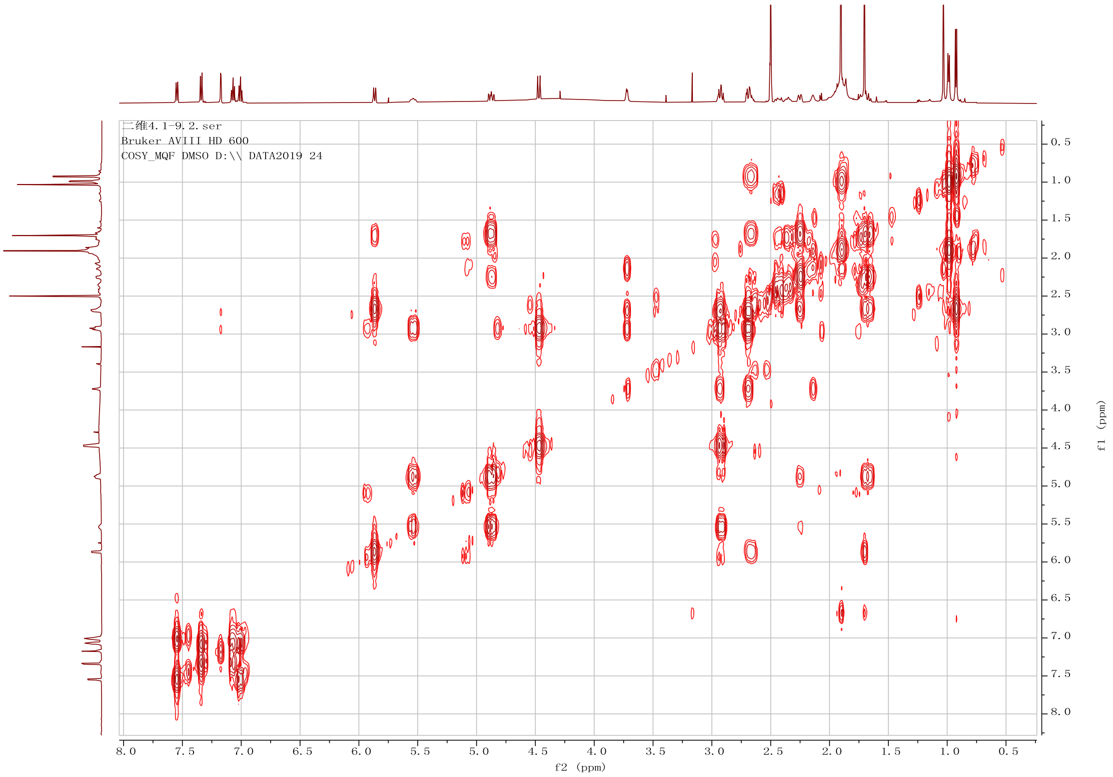

Figure S28: ROESY spectrum of **3**

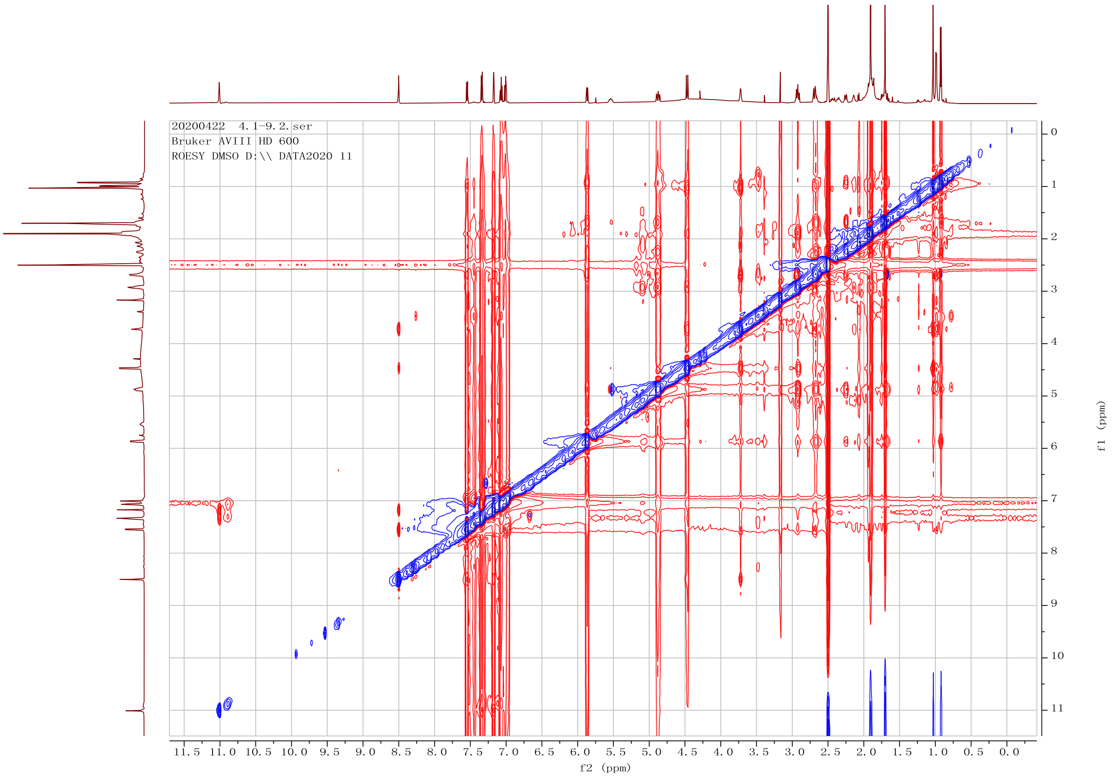

Figure S29: HR-ESIMS spectrum of **3**

Compod 3, ESI+, $[M+H]^+$ *m/z* 589.32963, $C_{34}H_{40}N_2O_7$

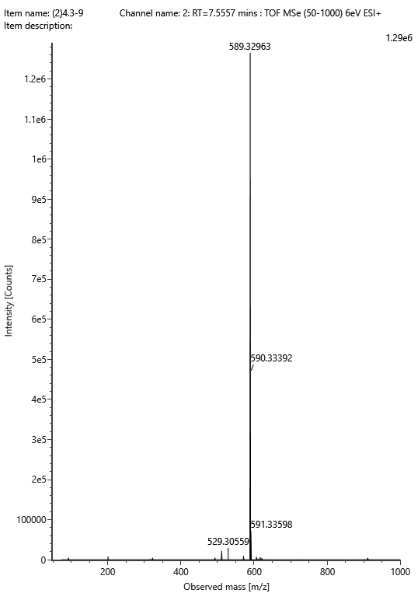

MS: 7.55 min, $[M+H]^+$ *m/z* 589.32963, TIC

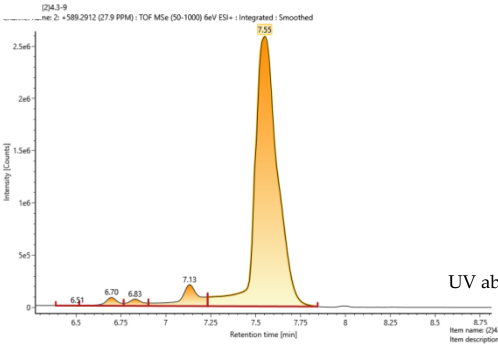

UV absorption: 221 and 255 nm

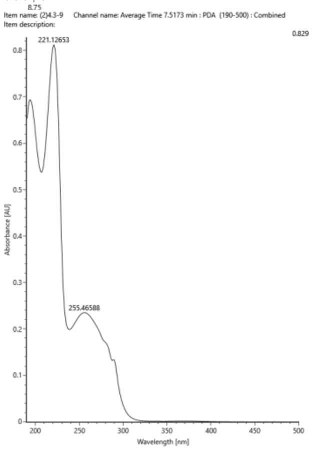

Composition	i-FIT confidence (%)	Intensity RMS (%)	Predicted m/z	m/z error (mDa)	measured m/z
$C_{34}H_{40}N_2O_7$	99.992767	0.87172	589.33211	-2.48	589.32963

Figure S30: ECD and UV spectrum of **3** (in MeOH)

TDDFT theory, ωB3LYP functional and 6-311G(d,p) level of theory, methanol as solvent for structural optimization, compound **3** has 13 conformations, of which 5 conformations have Boltzmann content >1%, listed in the table below. The calculated result of **3** is consistent with the experimental result, and the absolute configuration of **3** is determined as shown in the right figure.

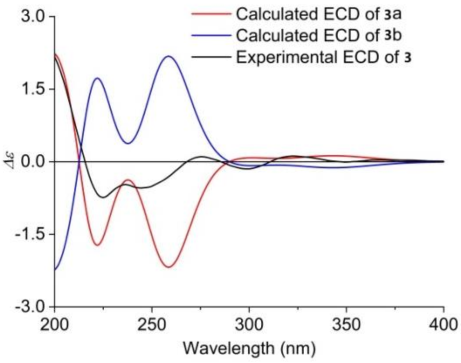

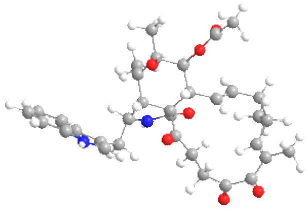	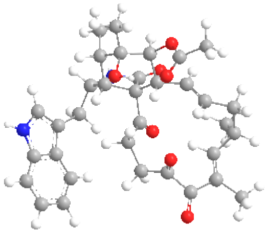	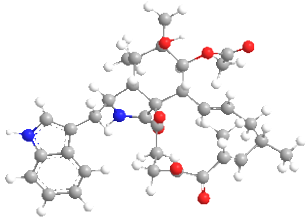
C1 (42.48%)	C2 (34.12%)	C3 (15.45%)
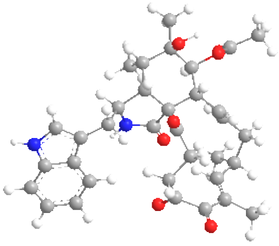	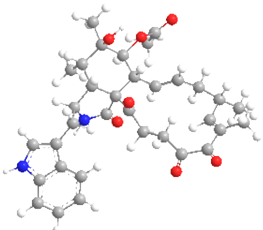	
C4 (3.78%)	C5 (3.23%)	

Figure S31 ^{1}H NMR (600 MHz, DMSO-*D6*) spectrum of **4**

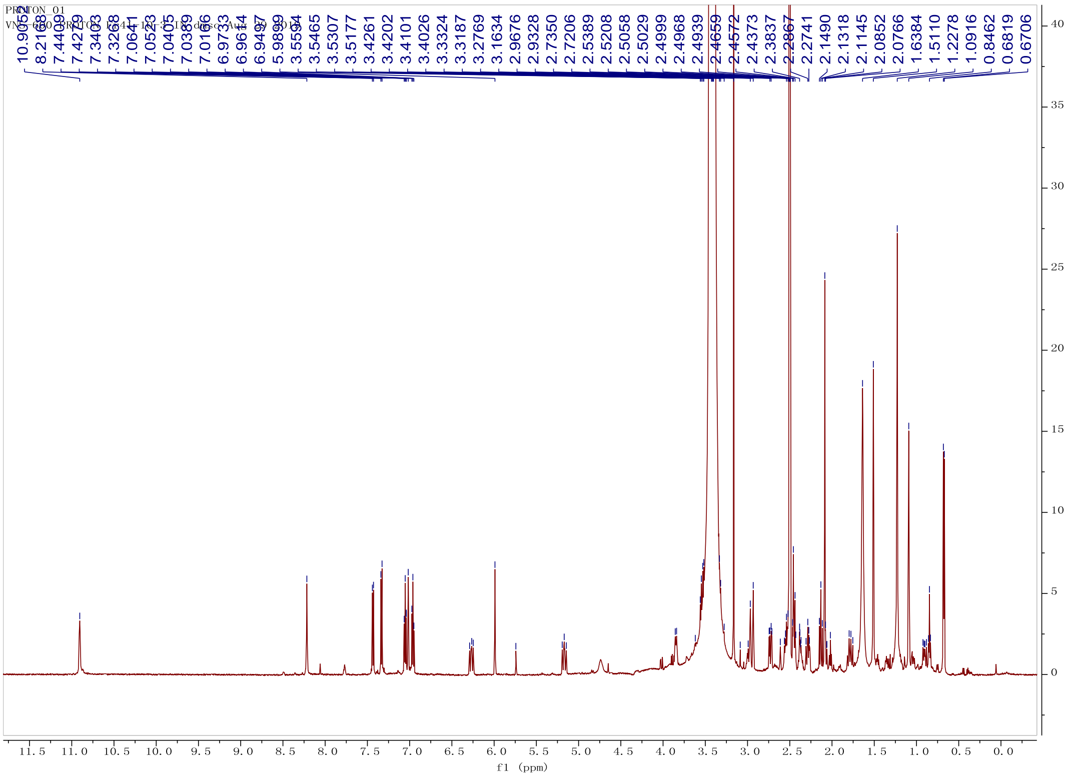

Figure S32: ^{13}C NMR (150 MHz, DMSO-*D6*) spectrum of **4**

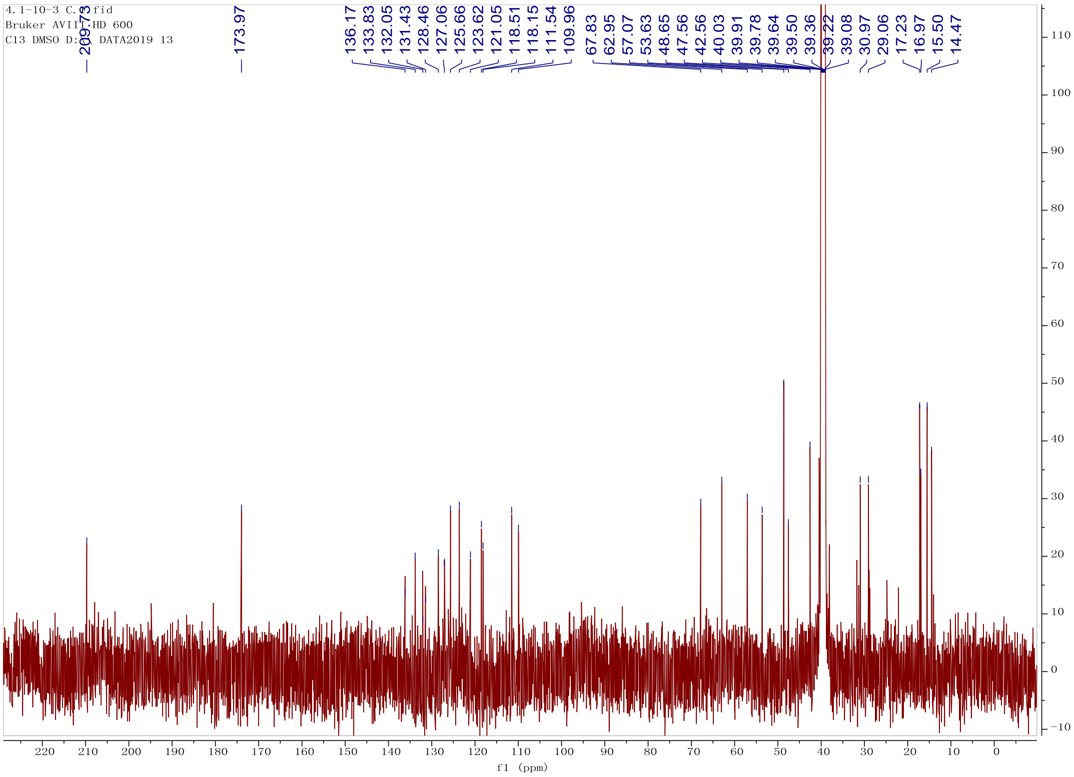

Figure S33: HSQC spectrum of **4**

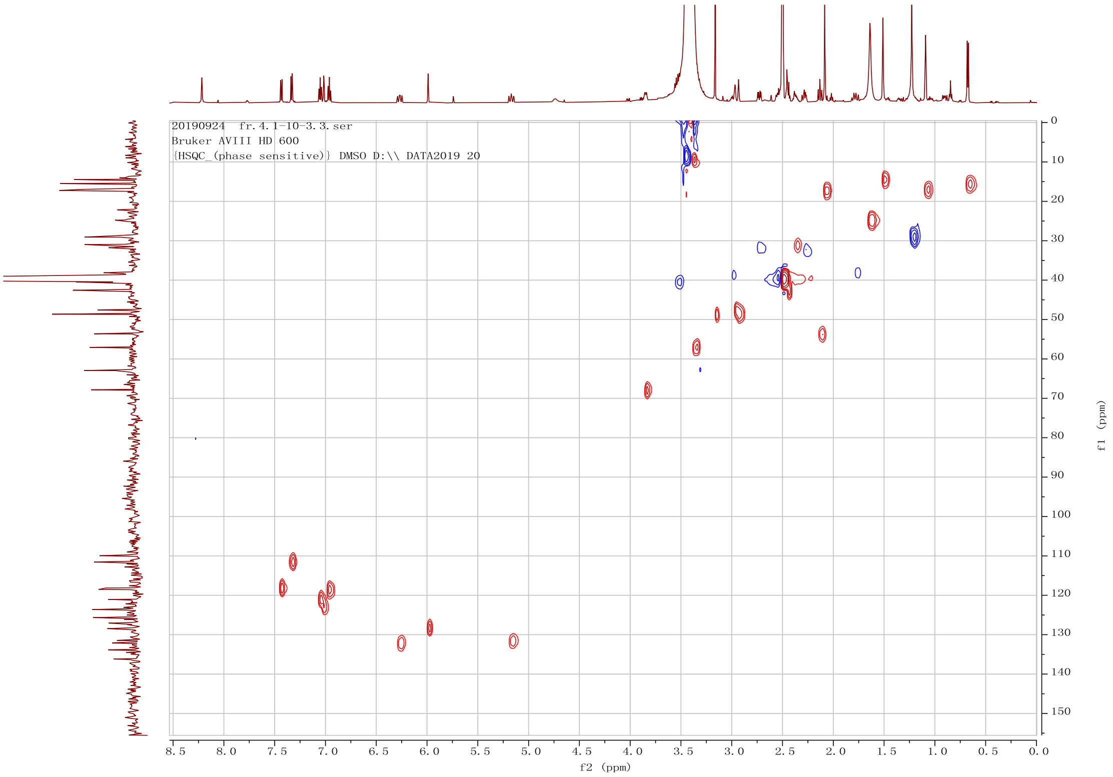

Figure S34: HMBC spectrum of **4**

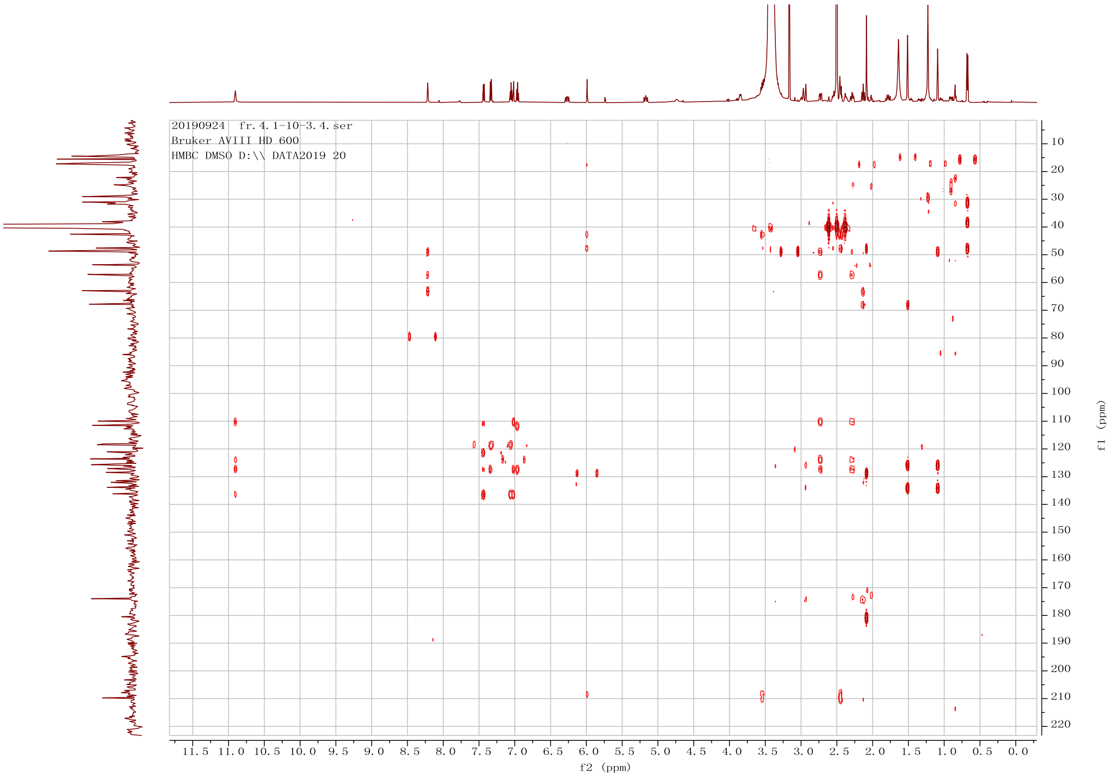

Figure S35: ^{1}H-^{1}H COSY spectrum of **4**

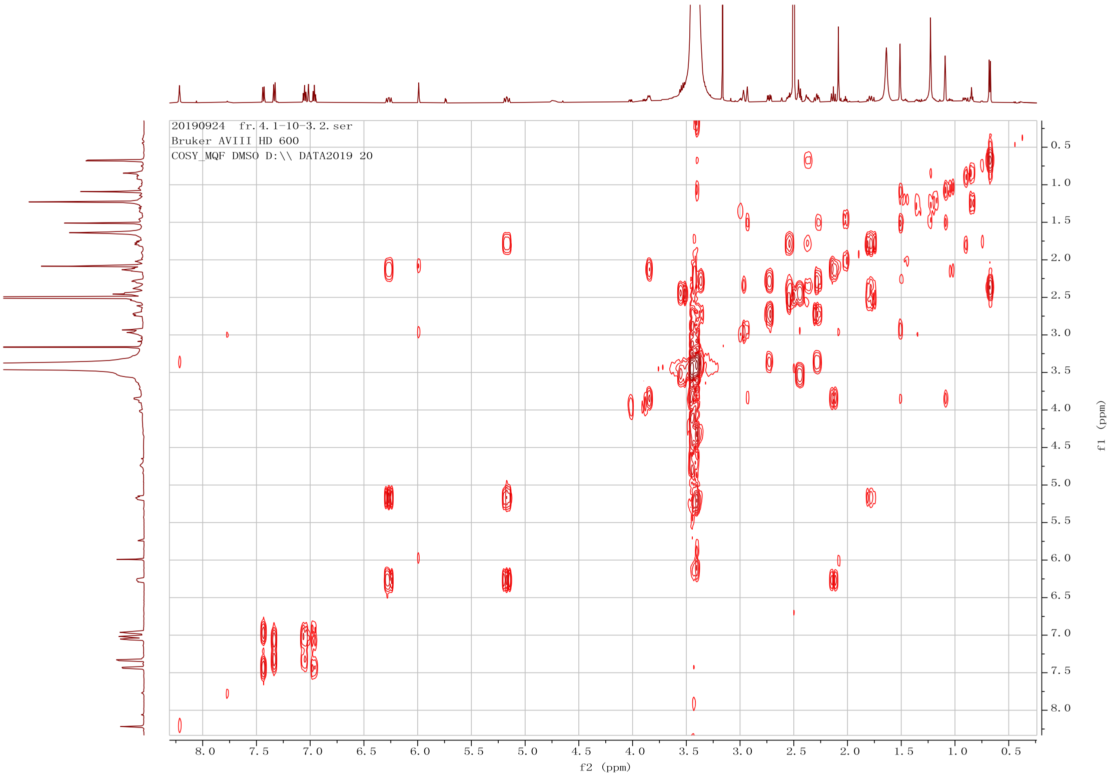

Figure S36: TOCSY spectrum of **4**

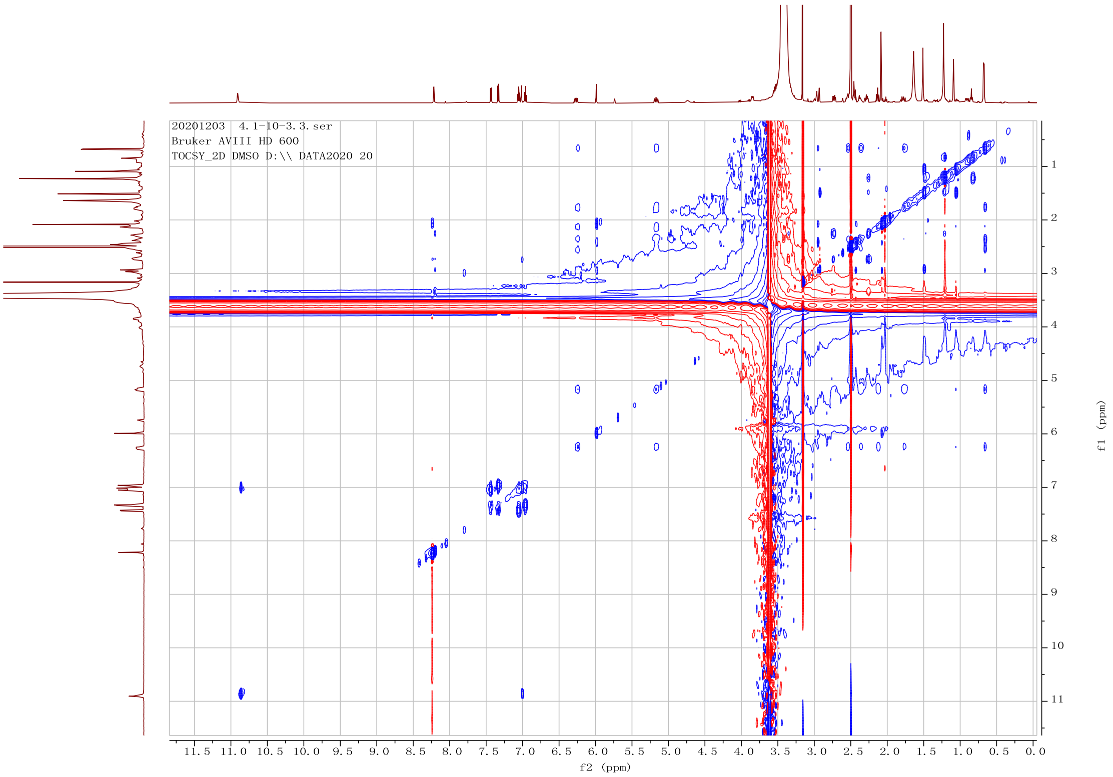

Figure S37: NOESY spectrum of **4**

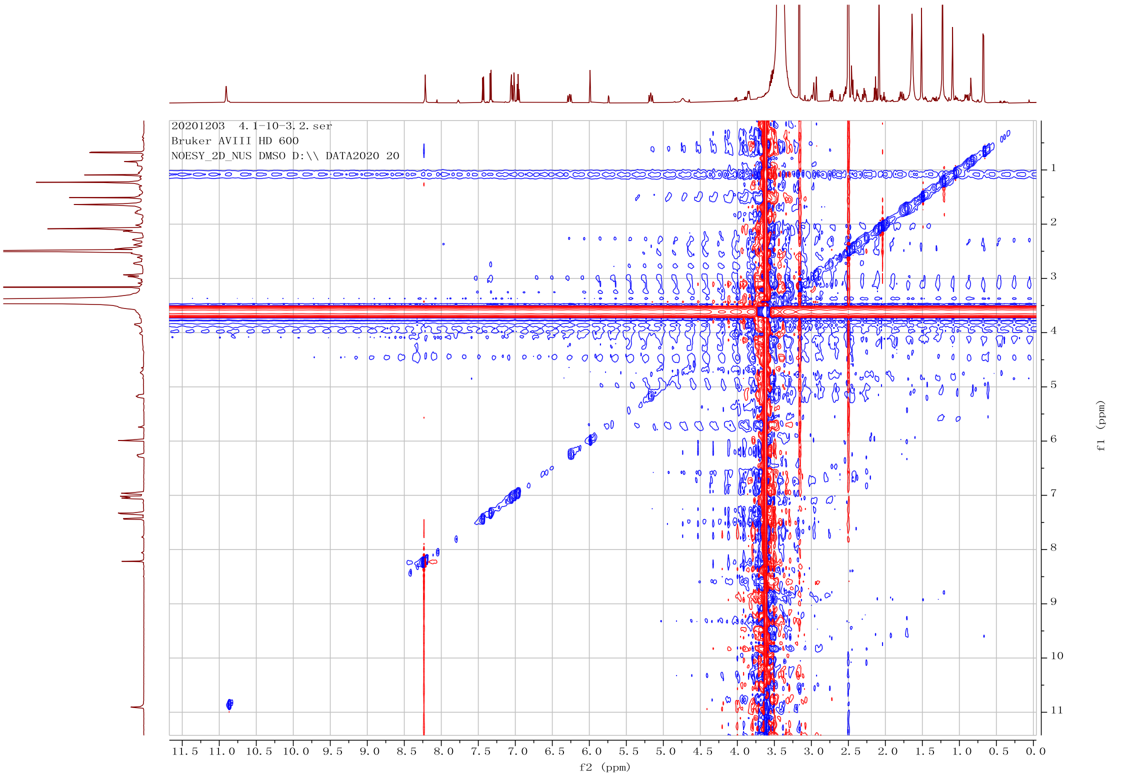

Figure S38: HR-ESI-MS spectrum of **4**

Compd 4 ESI+, [M+H]+ m/z 513.27413, t_R=3.42 min

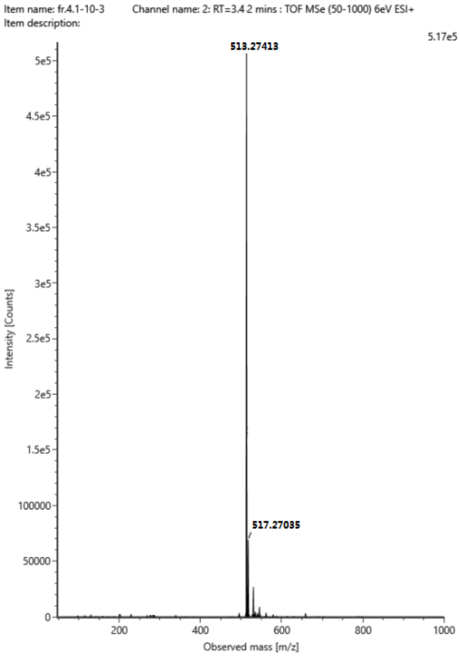

Compd **4**. 513.2741, t_R = 3.43 min

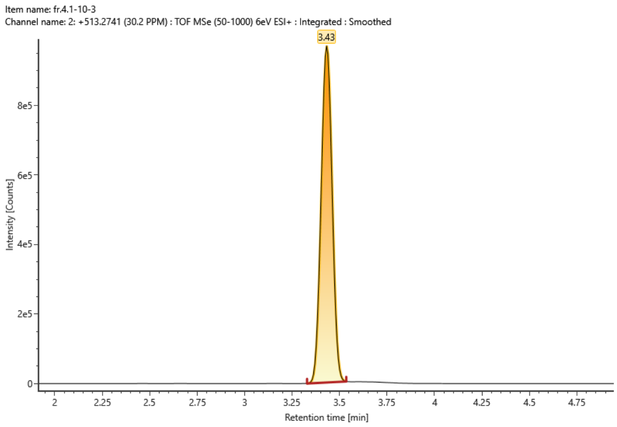

Results (1 found)

	Composition	i-FIT Confidence (%)	m/z RMS (PPM)	Intensity RMS (%)	Predicted m/z	m/z error (PPM)	m/z error (mDa)	DBE
1	C32H36N2O4	99.992657	1.325362	0.683619	513.274784	-1.276882	-0.654104	16.000000

Figure S39: ECD and UV spectrum of **4**

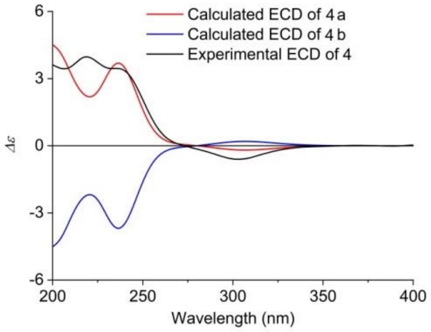

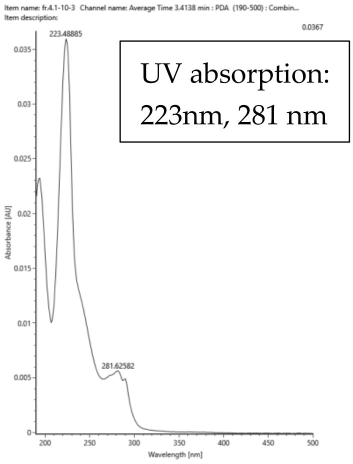

TDDFT theory, ωB3LYP functional and 6-311G(d,p) level of theory, methanol as solvent for structural optimization, compound **4** have 14 conformations, of which 8 conformations have Boltzmann content >1%, listed in the table below. The calculated result of **4** is consistent with the experimental result, and the absolute configuration of **4** is confirmed as shown in the figure above.

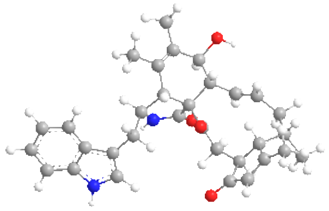	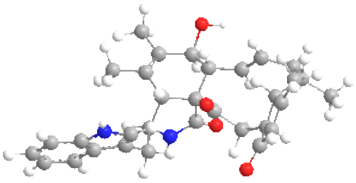	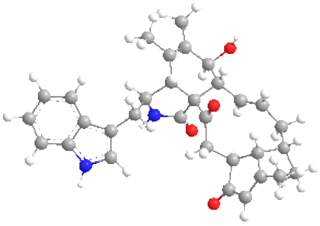
C1 (35.33%)	C2 (23.15%)	C3 (17.16%)
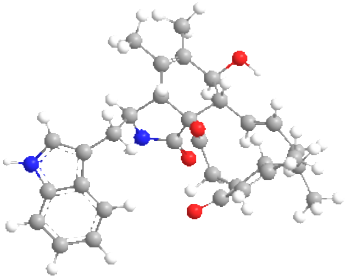	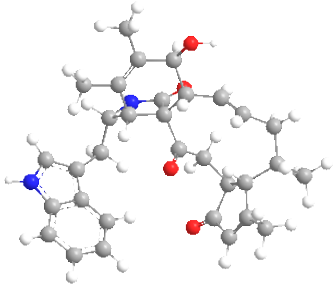	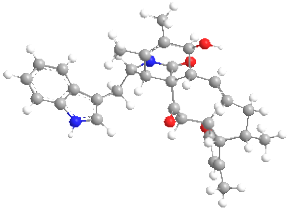
C4 (9.64%)	C5 (6.10%)	C6 (4.61%)
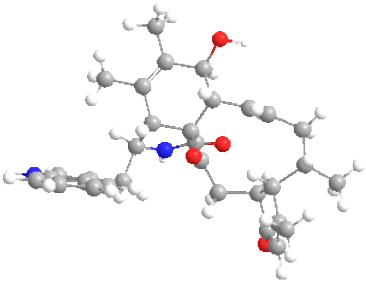	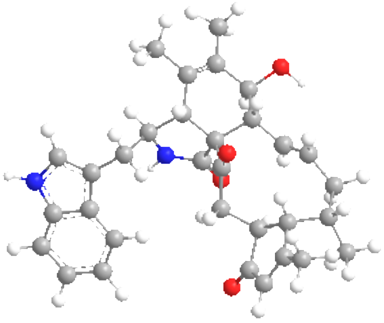	
C7 (2.01%)	C8 (1.87%)	

Figure S40: ^{1}H NMR (600 MHz, DMSO-*d6*) spectrum of **5**

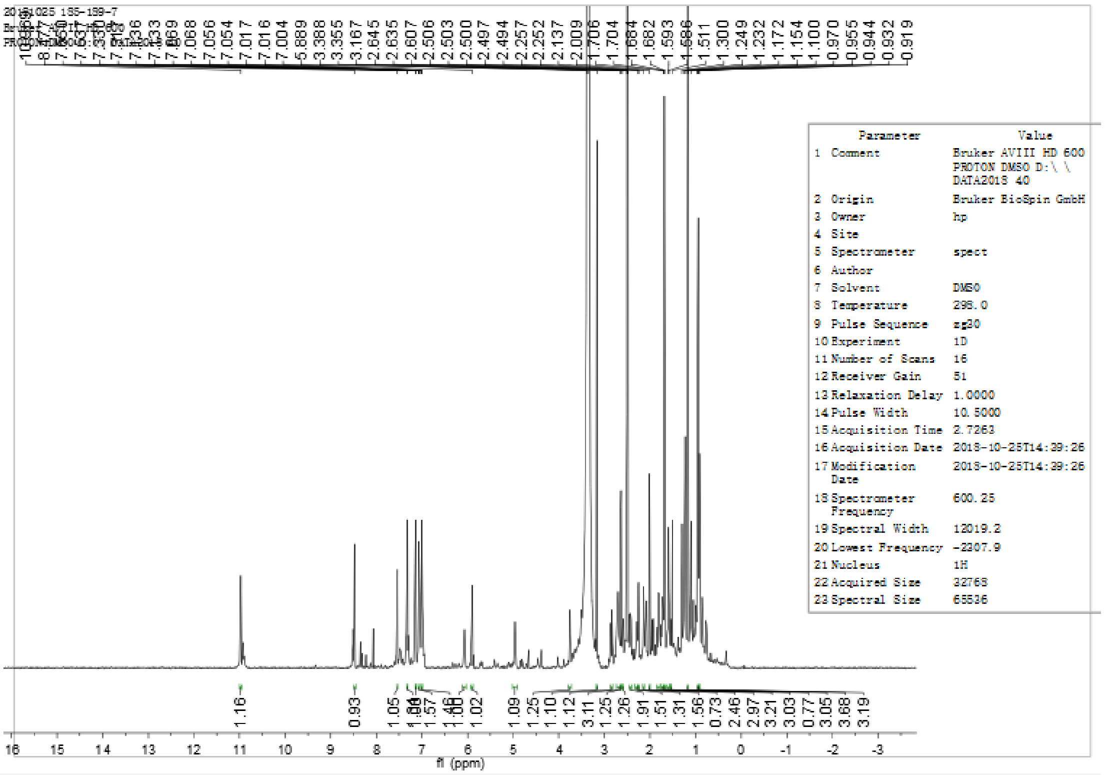

Figure S41:^{13}C NMR (150 MHz, DMSO-*d6*) spectrum of **5**

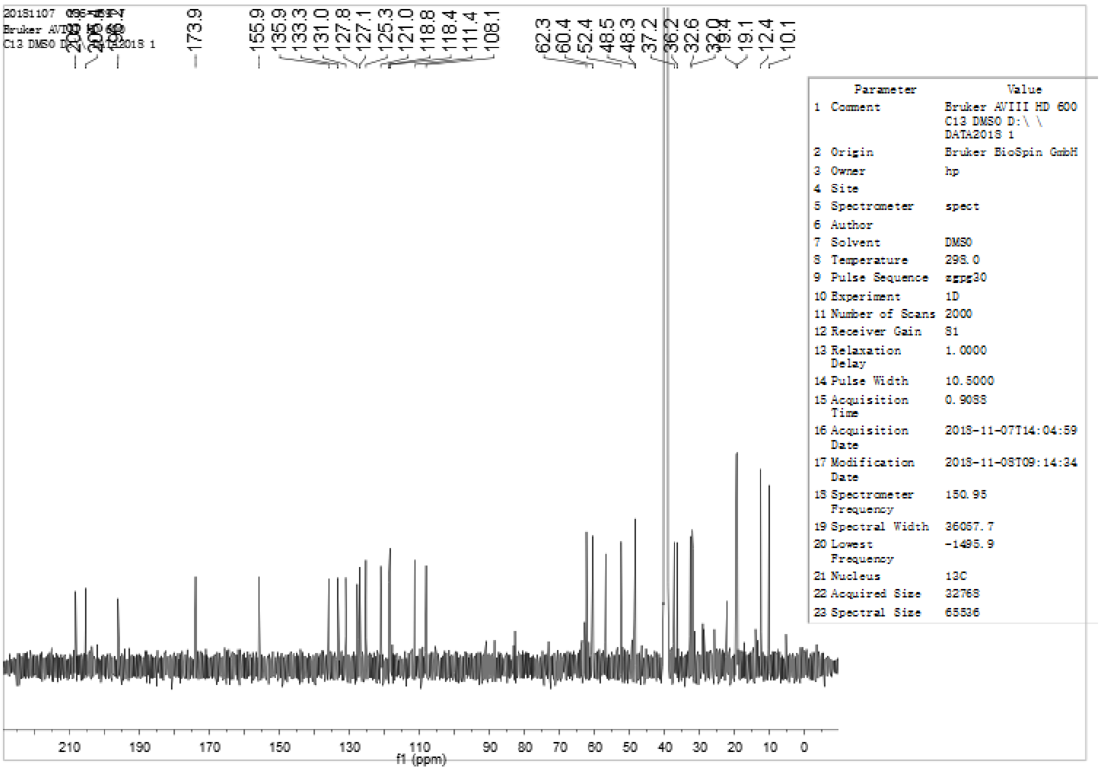

Figure S42: ^{1}H NMR (600 MHz, DMSO-*d6*) spectrum of **6**

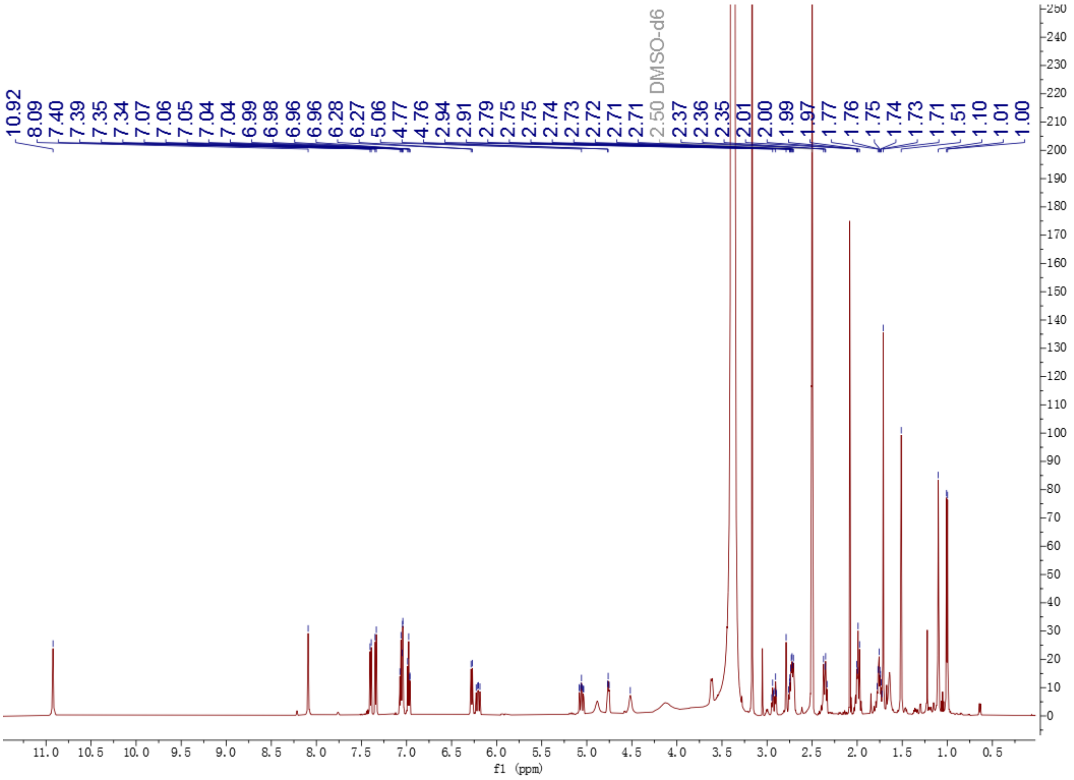

Figure S43:^{13}C NMR (150 MHz, DMSO-*d6*) spectrum of **6**

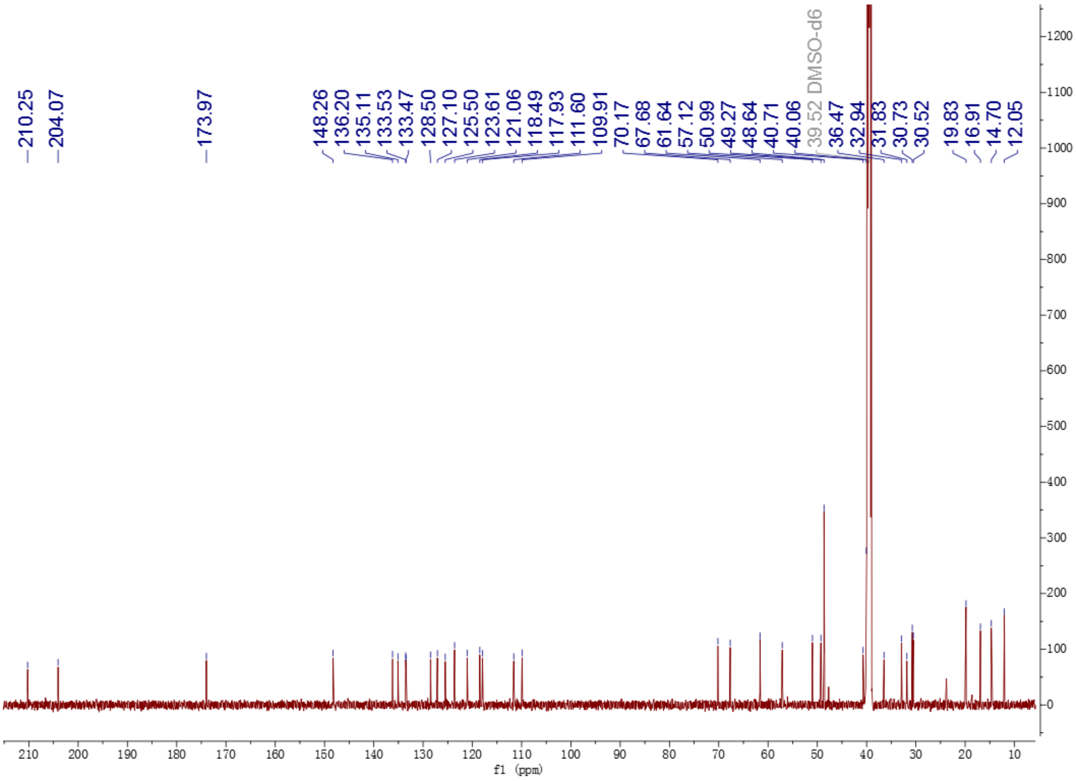

Figure S44: ^{1}H NMR (600 MHz, DMSO-d6) spectrum of **7**

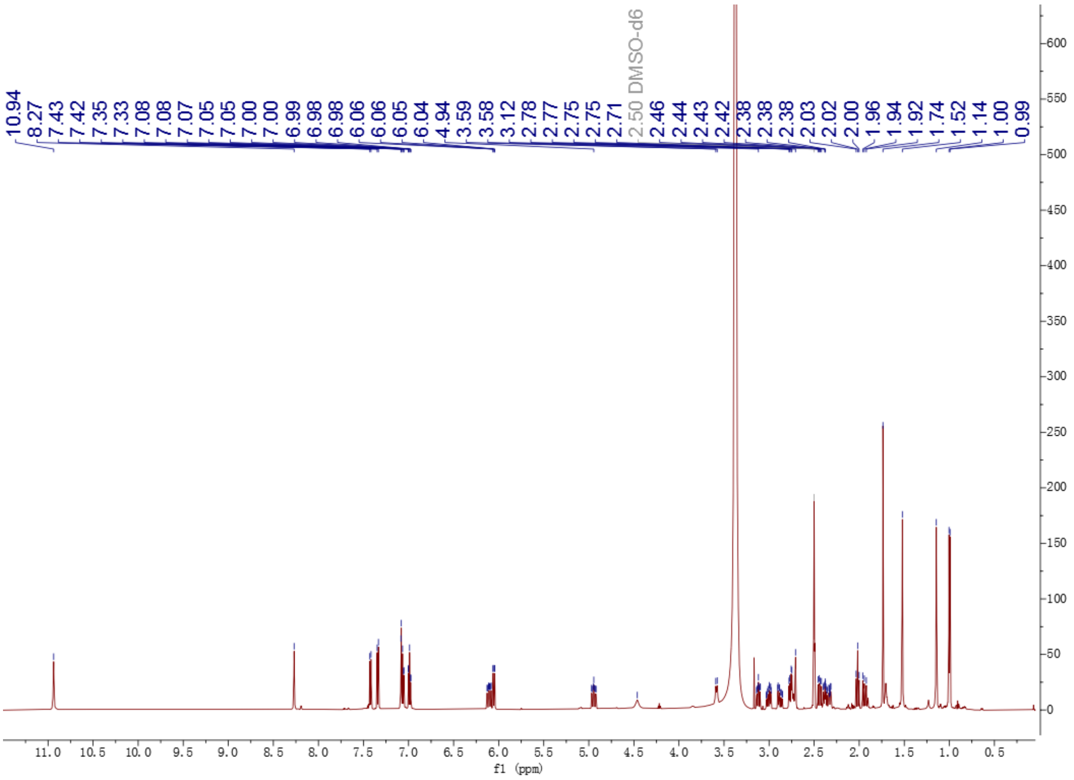

Figure S45:^{13}C NMR (150 MHz, DMSO-d6) spectrum of **7**

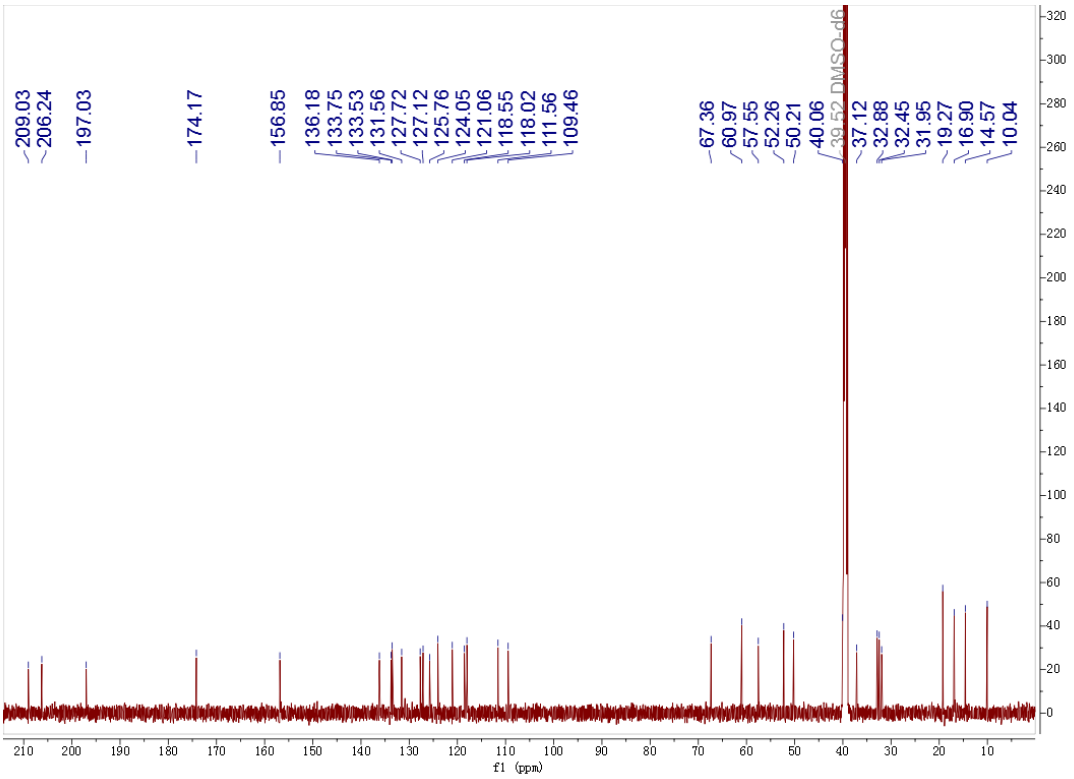

Figure S46: ^{1}H NMR (600 MHz, DMSO-*d6*) spectrum of **8**

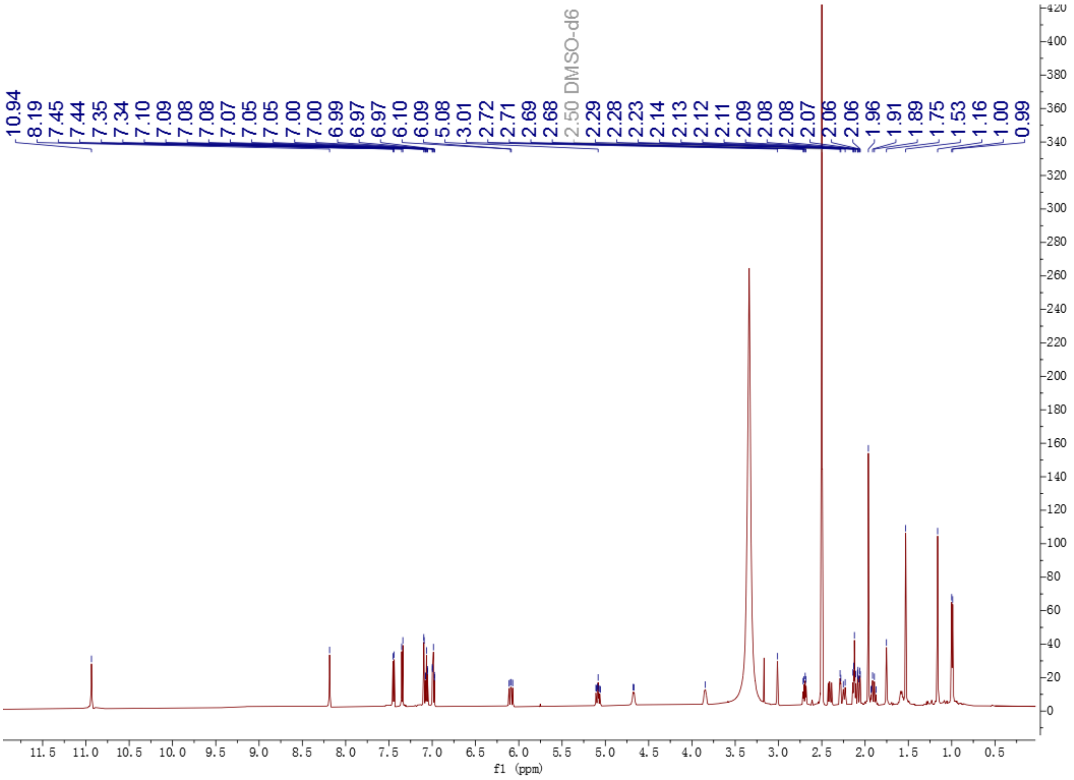

Figure S47: ^{13}C NMR (150 MHz, DMSO-*d6*) spectrum of **8**

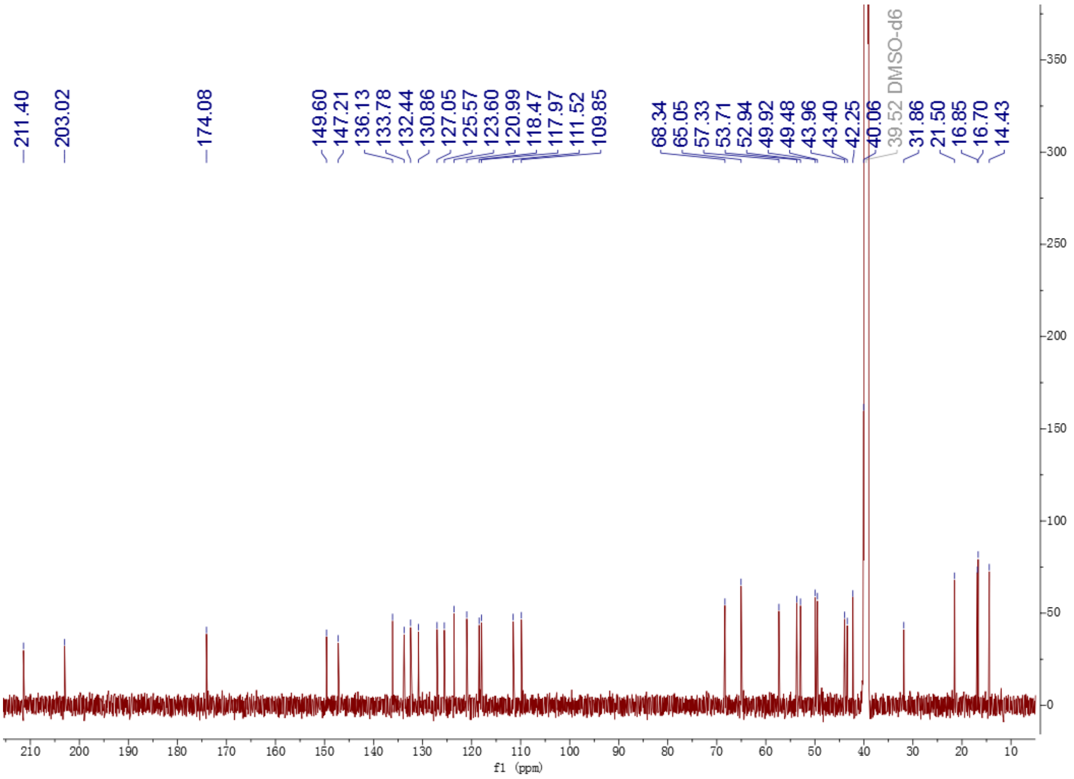

Figure S48: ^{1}H NMR (600 MHz, DMSO-*d6*) spectrum of **9**

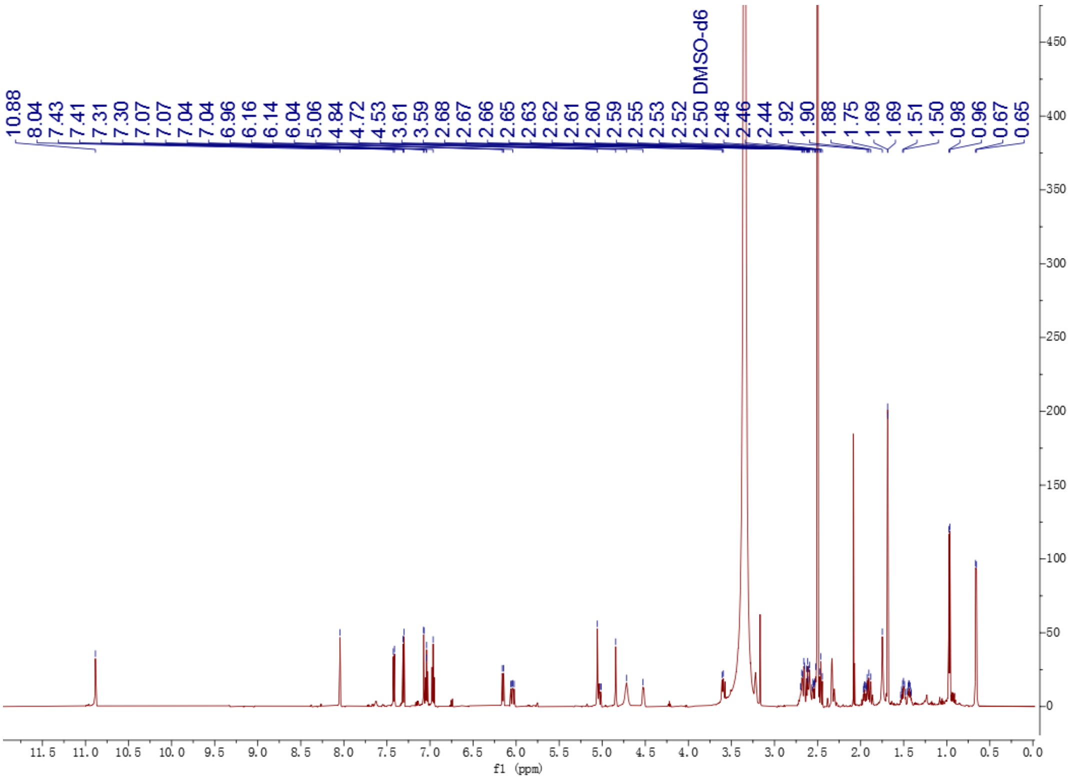

Figure S49: ^{13}C NMR (150 MHz, DMSO-*d6*) spectrum of **9**

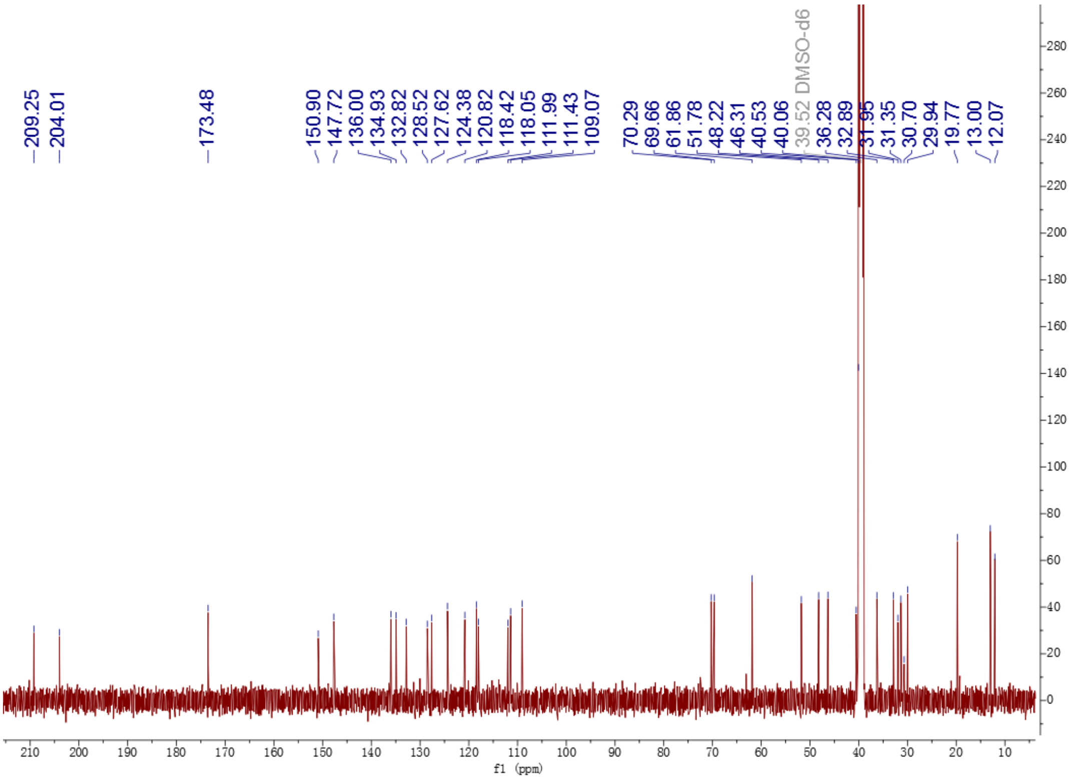

Figure S50: ^{1}H NMR (600 MHz, DMSO-*d6*) spectrum of **10**

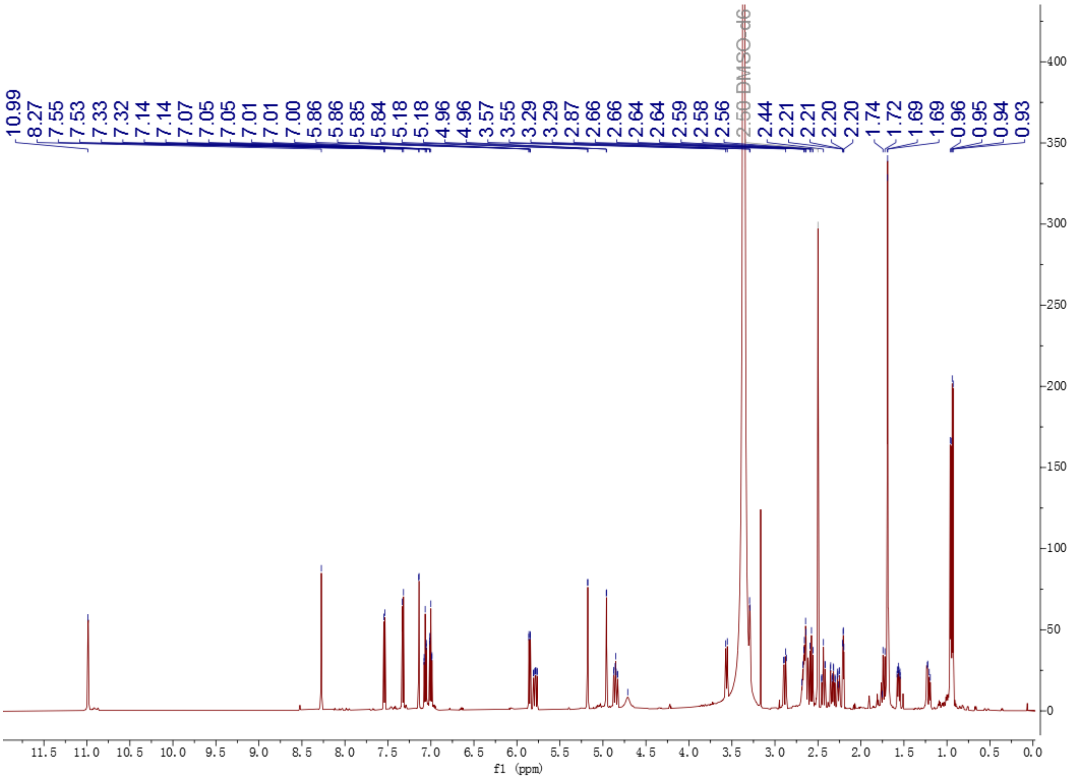

Figure S51:^{13}C NMR (150 MHz, DMSO-*d6*) spectrum of **10**

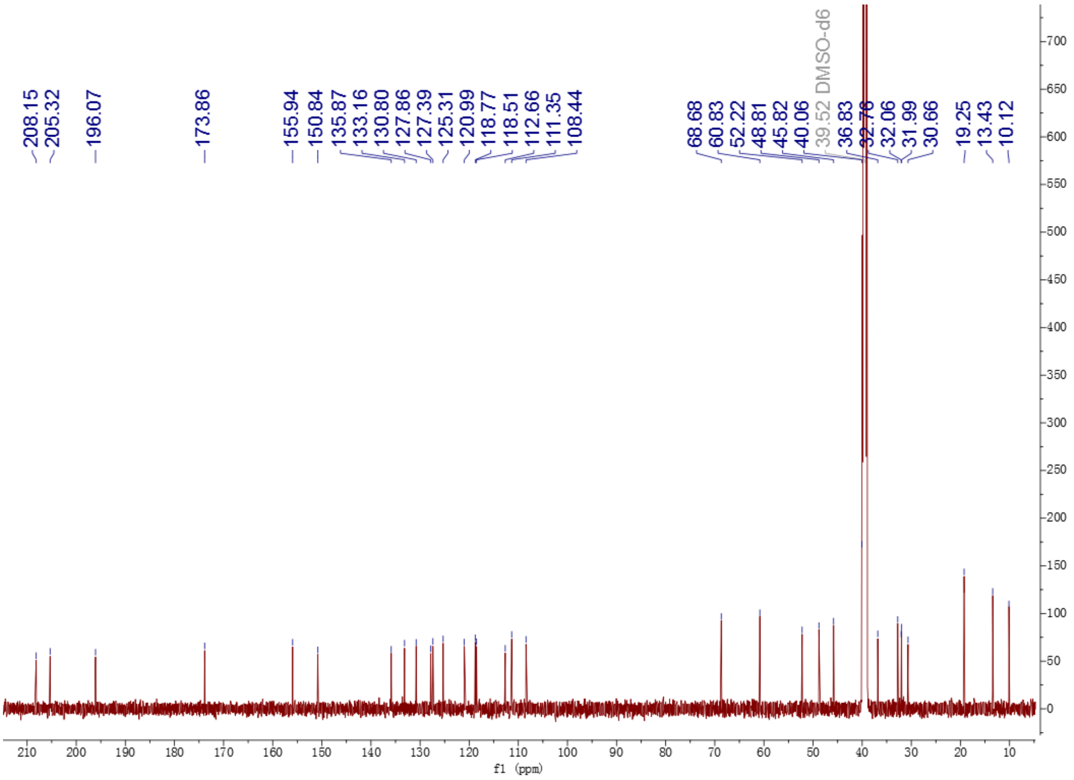

Figure S52: ^{1}H NMR (600 MHz, DMSO-*d6*) spectrum of **11**

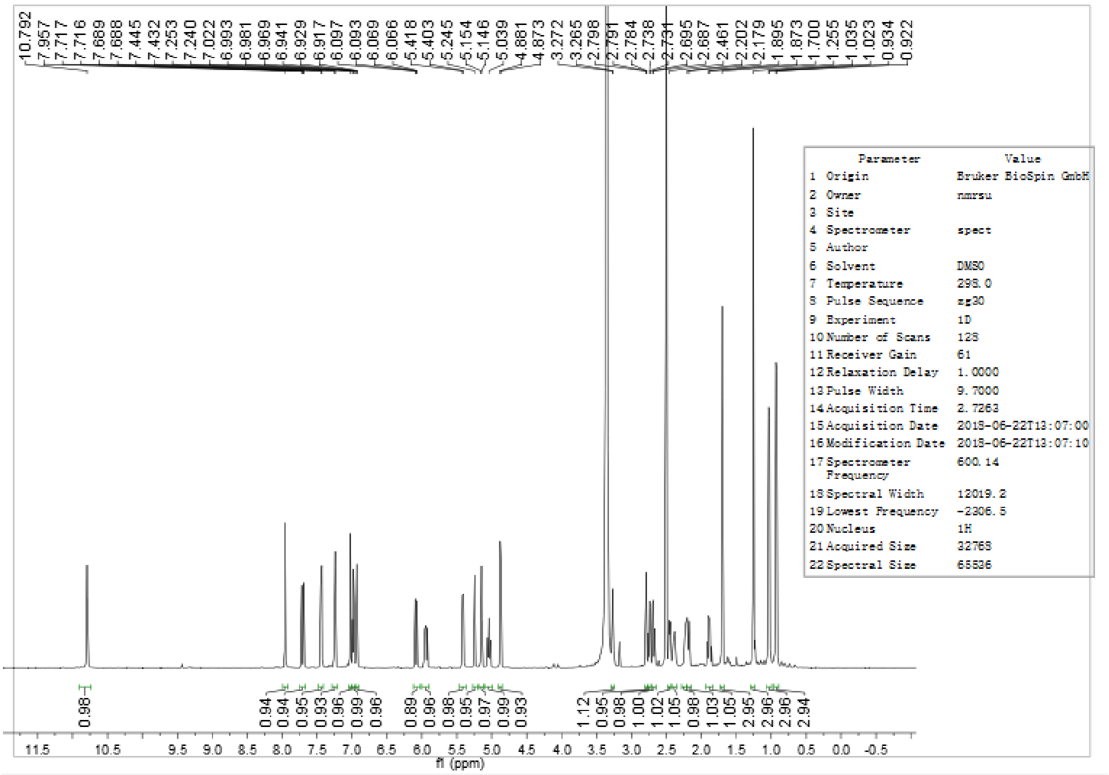

Figure S53: ^{13}C NMR (150 MHz, DMSO-*d6*) spectrum of **11**

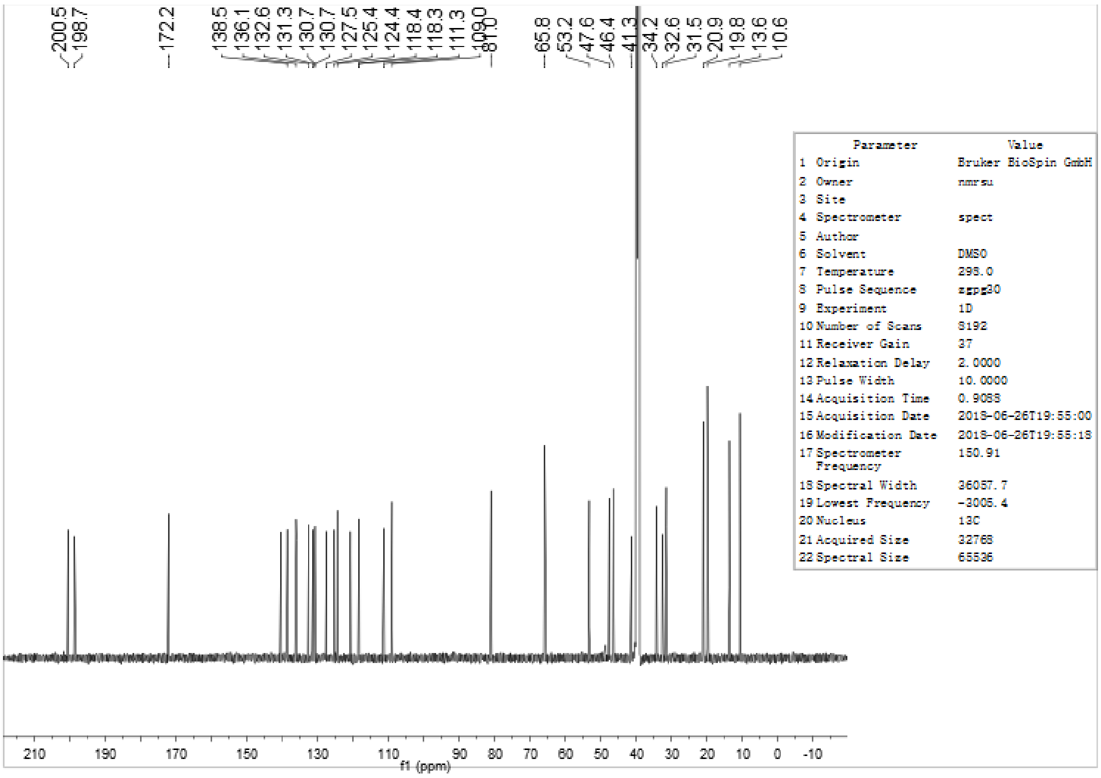

Figure S54: ^{1}H NMR (600 MHz, DMSO-*d6*) spectrum of **12**

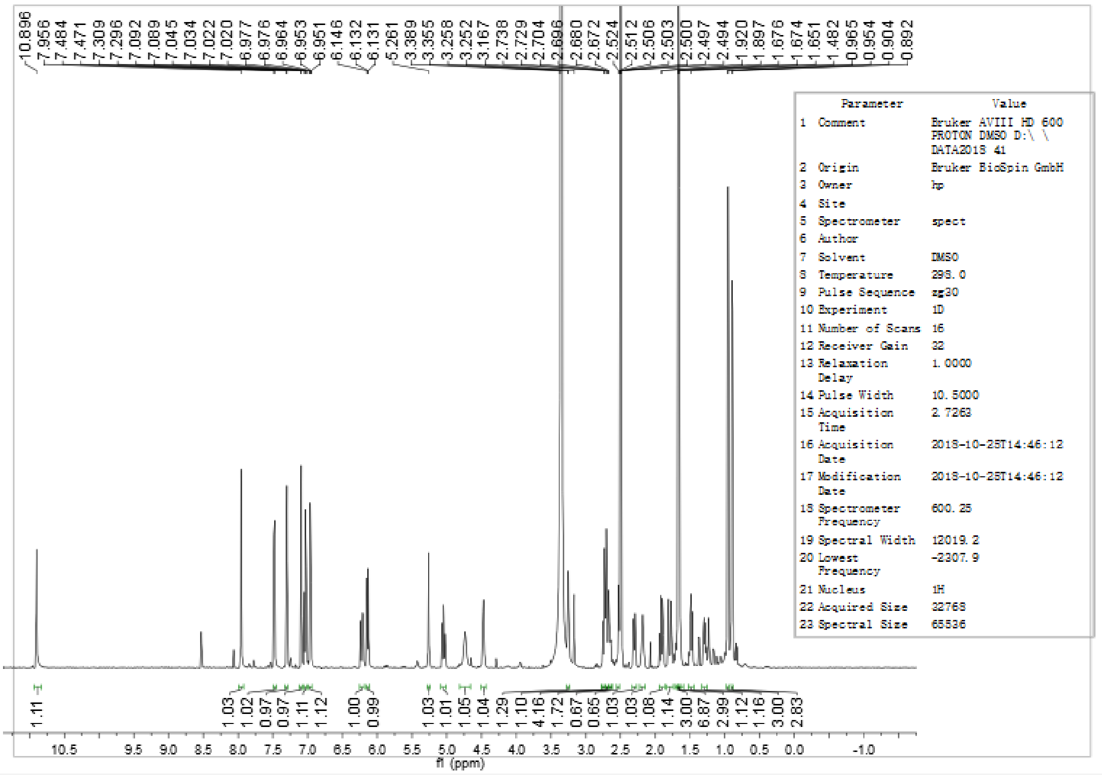

Figure S55: ^{13}C NMR (150 MHz, DMSO-*d6*) spectrum of **12**

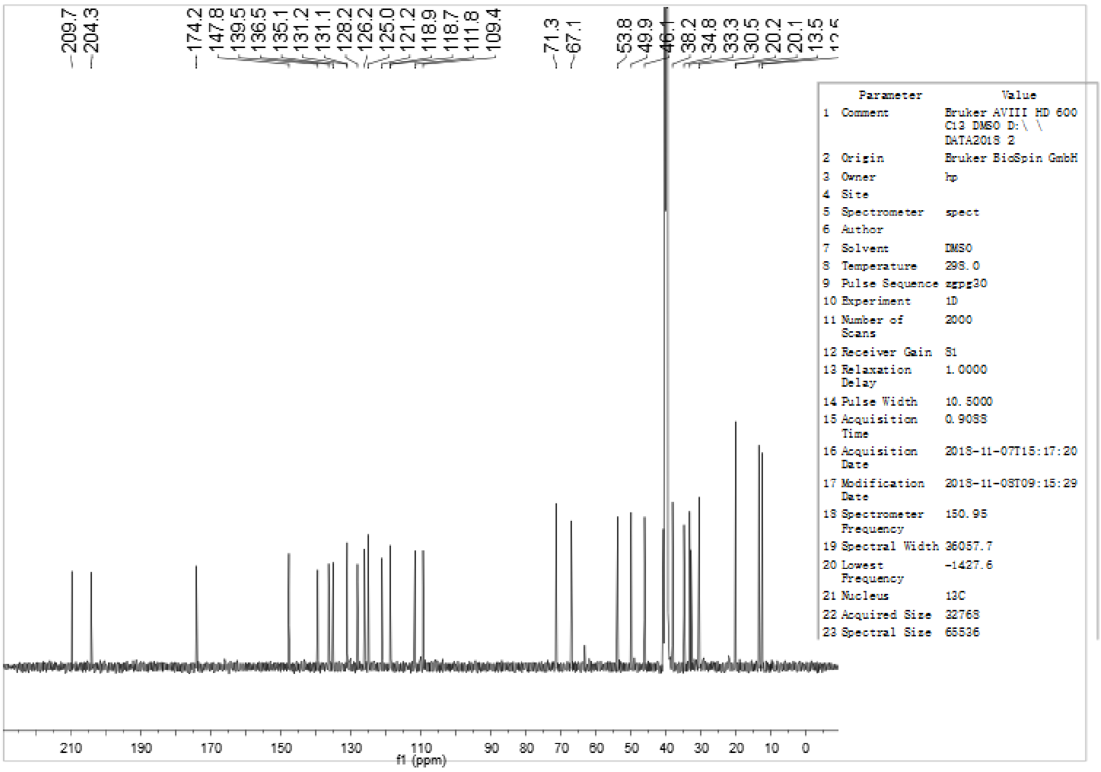

Figure S56: ^{1}H NMR (600 MHz, DMSO-*d6*) spectrum of **13**

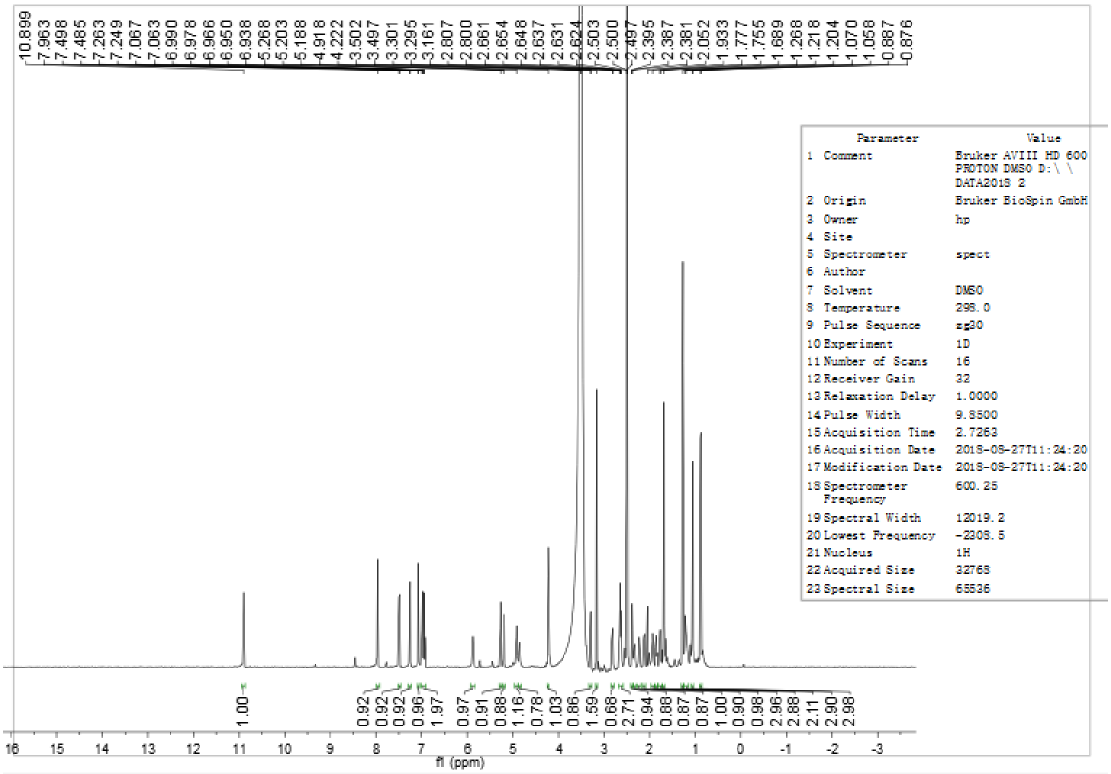

Figure S57:^{13}C NMR (150 MHz, DMSO-*d6*) spectrum of **13**

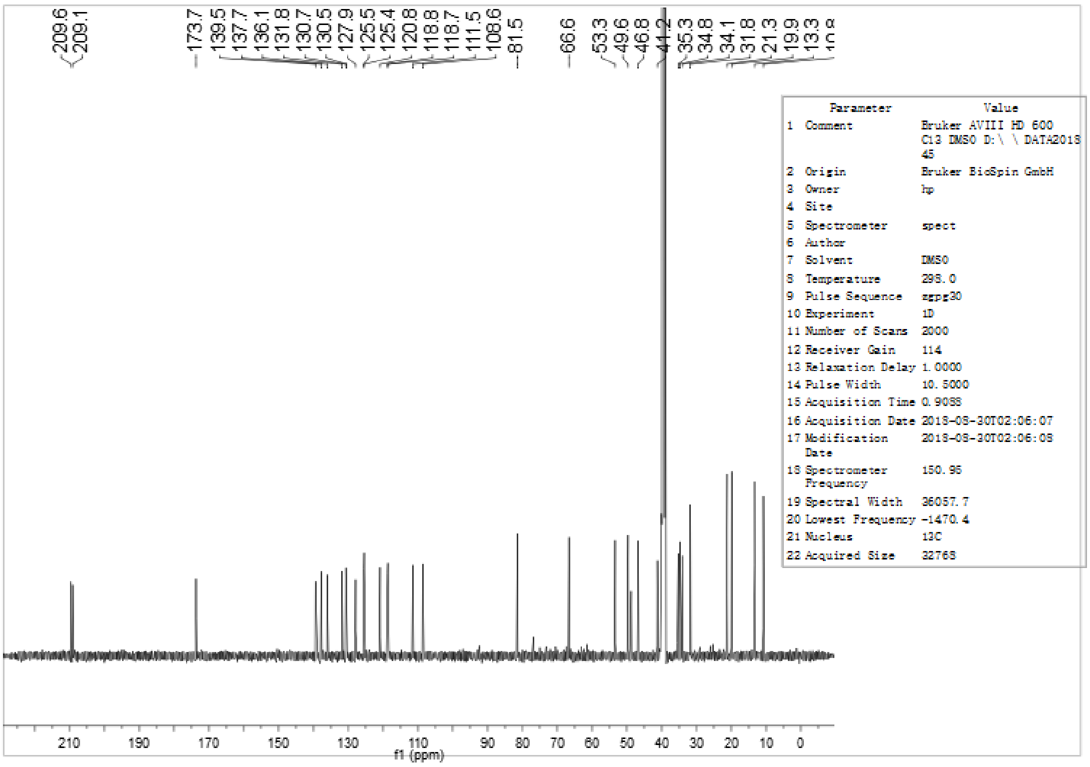

Figure S58: ^{1}H NMR (600 MHz, DMSO-*d6*) spectrum of **14**

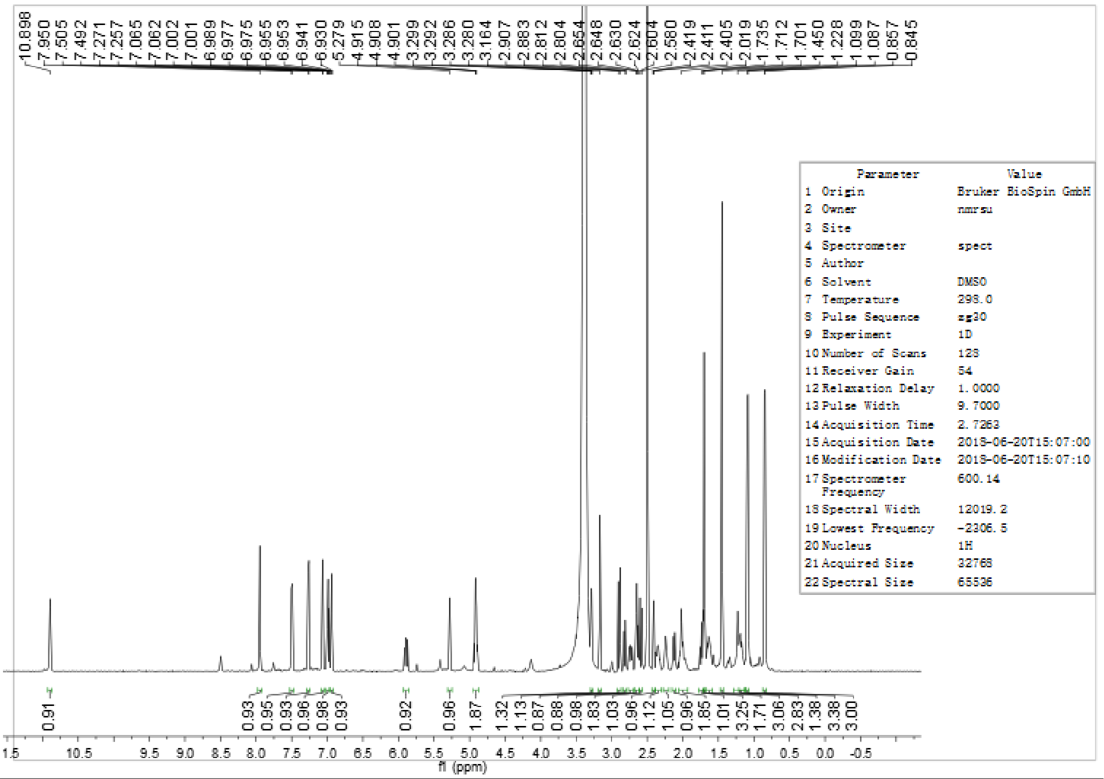

Figure S59:^{13}C NMR (150 MHz, DMSO-*d6*) spectrum of **14**

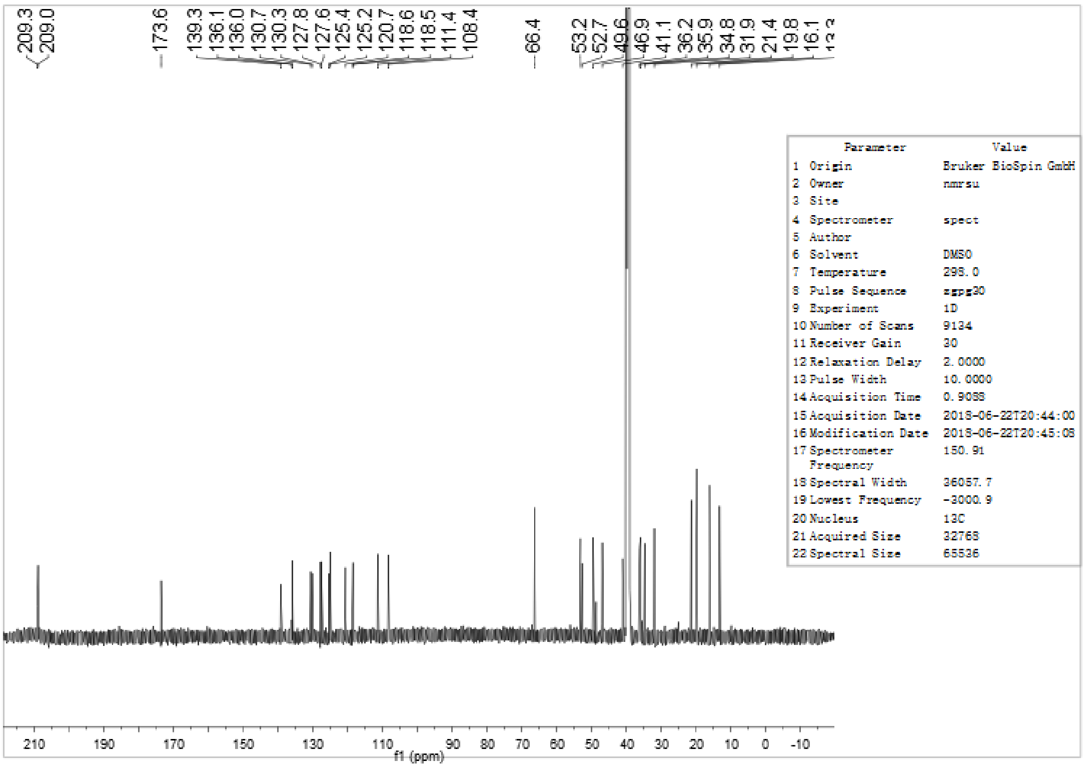

Figure S60: ^{1}H NMR (600 MHz, DMSO-*d6*) spectrum of **15**

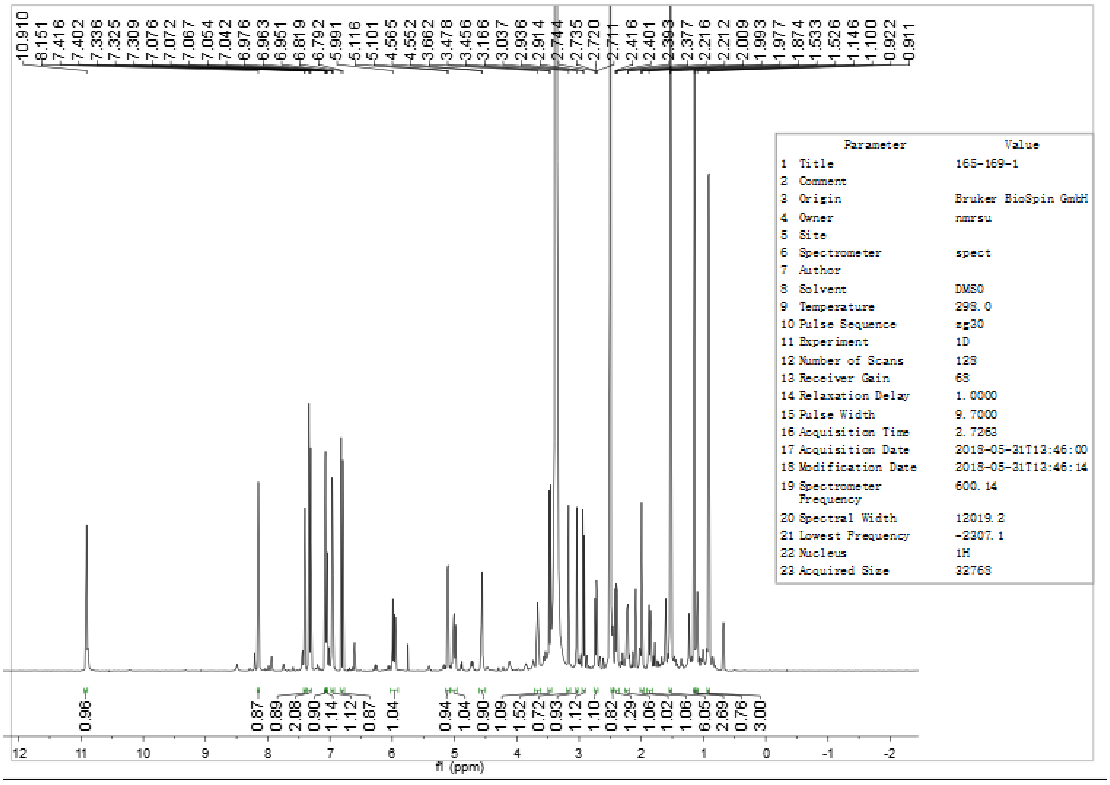

Figure S61: ^{13}C NMR (150 MHz, DMSO-*d6*) spectrum of **15**

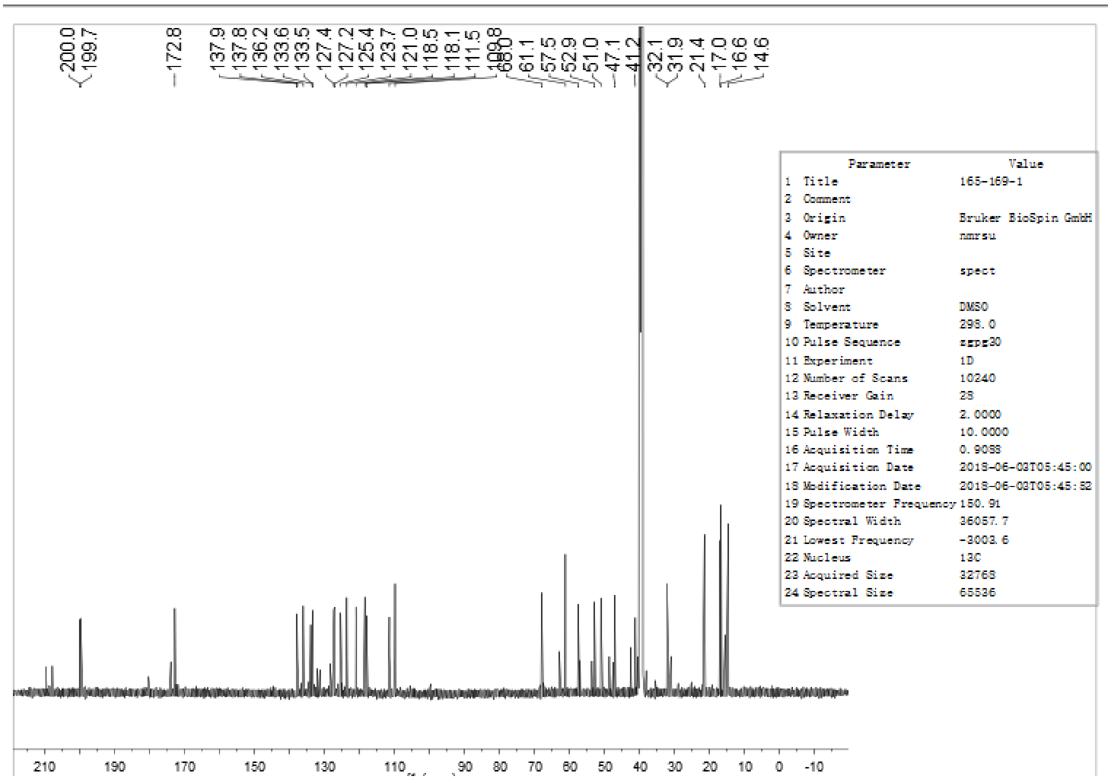

Figure S62: ¹H NMR (600 MHz, DMSO-*d6*) spectrum of **16**

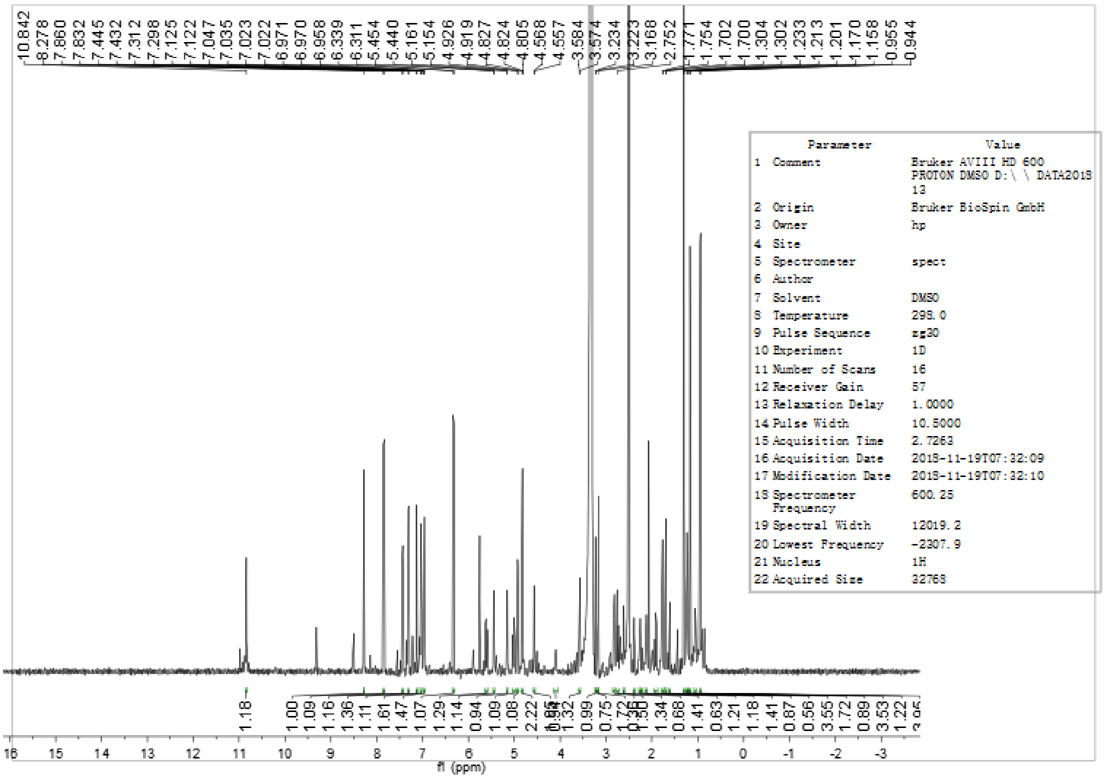

Figure S63:¹³C NMR (150 MHz, DMSO-*d6*) spectrum of **16**

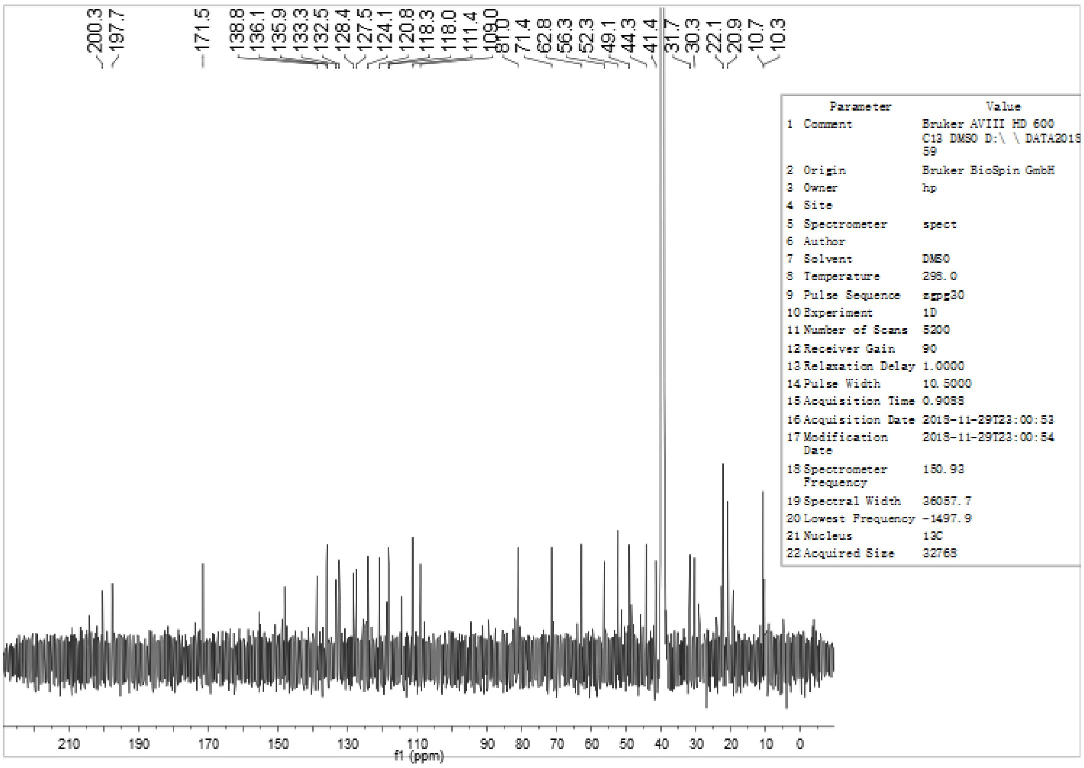

Supplement: Supplementary file 1 [file marinedrugs-19-00438-s001.zip › marinedrugs-1317021-supplementary.pdf]
